# Synthetic Biomimetic Liposomes Harness Efferocytosis Machinery for Highly Efficient Macrophages‐Targeted Drug Delivery to Alleviate Inflammation

**DOI:** 10.1002/advs.202308325

**Published:** 2024-05-24

**Authors:** Run Han, Zhengyu Ren, Qi Wang, Haidong Zha, Erjin Wang, Mingyue Wu, Ying Zheng, Jia‐Hong Lu

**Affiliations:** ^1^ State Key Laboratory of Quality Research in Chinese Medicine Institute of Chinese Medical Sciences (ICMS) University of Macau Macau 999078 China; ^2^ State Key Laboratory of Organic Electronics and Information Displays & Institute of Advanced Materials (IAM) Nanjing University of Posts & Telecommunications Nanjing 210023 China; ^3^ Faculty of Health Sciences University of Macau Macau 999078 China; ^4^ Guangdong‐Hong Kong‐Macau Joint Lab on Chinese Medicine and Immune Disease Research University of Macau Macau 999078 China

**Keywords:** biomimetic, efferocytosis, inflammatory bowel disease (IBD), liposome, macrophage targeting

## Abstract

Macrophages play pivotal roles in the regulation of inflammatory responses and tissue repair, making them a prime target for inflammation alleviation. However, the accurate and efficient macrophages targeting is still a challenging task. Motivated by the efficient and specific removal of apoptotic cells by macrophages efferocytosis, a novel biomimetic liposomal system called Effero‐RLP (Efferocytosis‐mediated Red blood cell hybrid Liposomes) is developed which incorporates the membrane of apoptotic red blood cells (RBCs) with liposomes for the purpose of highly efficient macrophages targeting. Rosiglitazone (ROSI), a PPARγ agonist known to attenuate macrophage inflammatory responses, is encapsulated into Effero‐RLP as model drug to regulate macrophage functions in DSS‐induced colitis mouse model. Intriguingly, the Effero‐RLP exhibits selective and efficient uptake by macrophages, which is significantly inhibited by the efferocytosis blocker Annexin V. In animal models, the Effero‐RLP demonstrates rapid recognition by macrophages, leading to enhanced accumulation at inflammatory sites. Furthermore, ROSI‐loaded Effero‐RLP effectively alleviates inflammation and protects colon tissue from injury in the colitis mouse model, which is abolished by deletion of macrophages from mice model. In conclusion, the study highlights the potential of macrophage targeting using efferocytosis biomimetic liposomes. The development of Effero‐RLP presents novel and promising strategies for alleviating inflammation.

## Graphical abstract

Harnessing the efferocytosis machinery by which macrophages selectively capture apoptotic cells, a macrophage‐targeting biomimetic drug delivery system (Effero‐RLP) for inflammation targeting is developed by the hybrid of apoptotic red blood cell membranes and liposomes. In DSS‐induced colitis model, Effero‐RLP demonstrated selective and efficient uptake by macrophages, leading to enhanced accumulation at inflammatory sites and improved therapeutic effect of rosiglitazone.

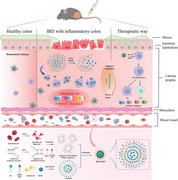

## 1 Introduction

Inflammation is a physiological response to many diseases that activate immune system for body's defense against infection and stimulus. It is the vital process that maintains tissue homeostasis following injury, by removing potential pathogens and beginning the process of repairing.^[^ ^1^ ^]^ Once the inflammation is unresolved or over activated, the acute and chronic inflammation would occur with pathogenicity. A vast range of diseases are caused by chronic inflammation including atherosclerosis, rheumatoid arthritis (RA), inflammatory bowel disease (IBD), nonalcoholic fatty liver disease, chronic obstructive pulmonary disease (COPD), asthma and others.^[^ ^2^ ^]^ In the process of inflammation, macrophages are essential for initiation, maintenance and resolution stages.^[^ ^3^ ^]^ It could quickly migrate and enrich into inflammatory sites, where performs the dual role including disease progression and tissue reparations.^[^ ^4^, ^5^ ^]^ Generally, macrophages can be polarized into pro‐inflammatory and anti‐inflammatory phenotypes. Pro‐inflammatory phenotype is response to produce inflammatory cytokines and chemokines that trigger tissue immune responses. Whereas anti‐inflammatory phenotype can release many anti‐inflammatory cytokines for tissue repairing, potential pathogens clearing and inflammatory microenvironment reprogramming.^[^ ^6^ ^]^ Accordingly, macrophages are central to inflammation and has emerged as potential targets of many inflammatory diseases.

The clearance of dead cells’ bodies and debris, particularly through phagocytosis by macrophages, terms as efferocytosis, plays an integral role in tissue repairing of inflammatory disease.^[^ ^7^ ^]^ During efferocytosis, macrophages efficiently clear excess apoptotic cells and release cytokines such as IL‐10 and TGF‐*β* to facilitate inflammation resolution.^[^ ^7^, ^8^, ^9^ ^]^ Upon the occurrence of cell apoptosis, the arrangement of lipid components in cell membrane undergoes alterations, leading to the redistribution of phosphatidylserine (PS) from its internal location to expose on the cell surface. In particular, the initialization of efferocytosis relies on the recognition of PS by macrophages and consequently stimulates phagocytosis of dead cells.^[^ ^10^, ^11^, ^12^ ^]^ This non‐inflammatory and non‐immunogenic manner provides a new inspiration for drug delivery system with macrophage targeting that is applied for anti‐inflammation.

The present study focuses on the development of biomimetic liposomes that harness efferocytosis machinery for inflammatory bowel disease (IBD) treatment. Macrophages play a crucial role in maintaining intestinal microenvironment homeostasis.^[^ ^13^ ^]^ In IBD, substantially increasing macrophages are activated and infiltrated into lamina propria of intestine. Notably, the recruited macrophages tend to polarize into pro‐inflammatory M1 phenotype, exacerbating inflammation while concurrently displaying reduced anti‐inflammatory M2 phenotype. This imbalance between M1 and M2 phenotypes disrupts intestinal immune homeostasis.^[^ ^14^, ^15^ ^]^ To enhance therapeutic efficiency, there is ongoing interest in developing biomimetic drug delivery systems for anti‐inflammation. For instance, β‐cyclodextrin nanoparticles coated with macrophage membrane have demonstrated great targeting and therapeutic effects for ulcerative colitis (UC) treatment.^[^ ^16^ ^]^ Additionally, liposomes fused with lipopolysaccharides (LPS)‐activated neutrophil membranes have been utilized to construct neutrophil liked nanoparticles capable of targeting intercellular adhesion molecule‐1 at inflammatory colon sites for alleviating colitis.^[^ ^17^ ^]^

Inspired by the macrophage targeting nature of apoptotic cells and the non‐immunogenic feature for efferocytosis, it could be speculated that apoptotic cell membrane can be prepared as biomimetic liposomes to facilitate macrophage targeting efficiency. In this study, RBCs were treated with hydrogen peroxide to induce apoptosis, and the resulting apoptotic RBC membranes were co‐extruded with liposomes to generate efferocytosis‐enhancing biomimetic liposomes (Effero‐RLP). Inflammatory stimuli induced the higher expression of nitric oxide synthase (iNOS) and oxidative stress enzymes in macrophages, as well as the release of pro‐inflammatory cytokines in the inflammatory colon lumen.^[^ ^18^, ^19^ ^]^ ROSI, an agonist of peroxisome proliferator activated receptor gamma (PPAR‐γ) with anti‐inflammatory effect was encapsulated into Effero‐RLP for IBD treatment.^[^ ^20^ ^]^ Collectively, efferocytosis based biomimetic nanoparticle was a promising way for anti‐inflammation.

## 2 Results

### 2.1 Preparation and Characterization of Effero‐RLP

**Figure 1:**
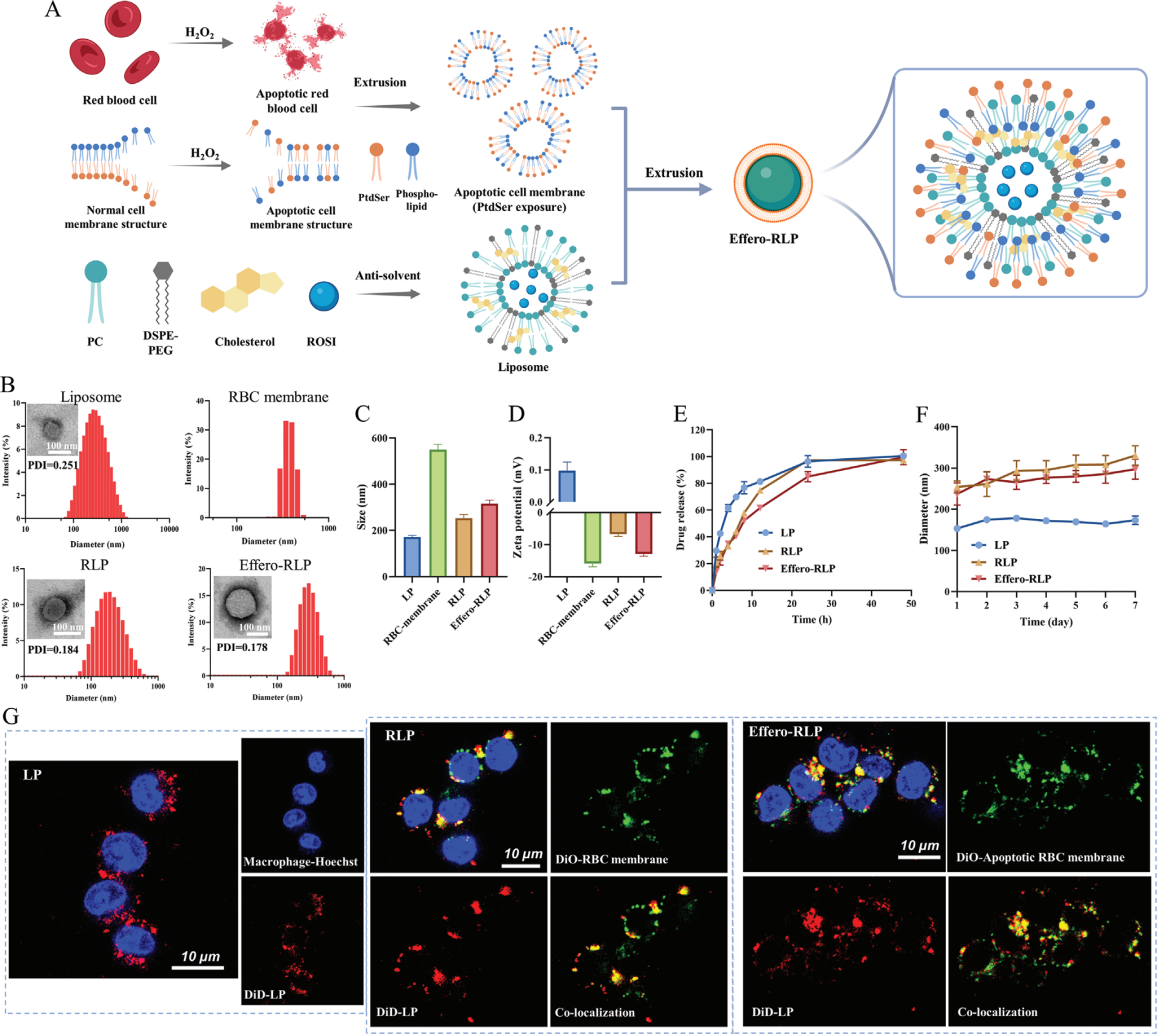
The preparation of LP, RLP and Effero‐RLP. A) The schematic of apoptotic RBC membrane preparation and the fusion with liposome particles. B) Size distribution of RBC membrane, LP, RLP and Effero‐RLP, and the particle structure of LP, RLP and Effero‐RLP. C) Hydrated particle size of liposome, RBC membrane, RLP and Effero‐RLP. D) Surface zeta potential of liposome, RBC membrane, RLP and Effero‐RLP. E) Drug release profile of liposome, RLP and Effero‐RLP. F) Stability of liposome, RLP and Effero‐RLP for 7 days storage. G) Co‐localization of DiD‐labeled liposomes and DiO‐labeled RBC membrane or apoptotic RBC membrane in RLP and Effero‐RLP.

The phagocytosis of apoptotic RBCs by macrophages was observed under microscope. It was evident that normal RBCs were not captured by macrophages, whereas the apoptotic RBCs were promptly recognized and engulfed by macrophages (Figure S1, Supporting Information). To achieve the effective macrophage targeting, apoptotic RBC membrane hybrid with liposomes were prepared. First, RBC was incubated with 1.25 mm H_2_O_2_ for 4 h to induce 60%–70% of cell apoptosis rate (Figure S2, Supporting Information). PS as a specific “eat‐me” signal of cell apoptosis was exposed on the surface of cell membrane, then led to efferocytosis by macrophages. Reaping the advantages of efferocytosis, apoptotic RBC membranes were hybrid with liposomes to prepare biomimetic liposomes for macrophage targeting as **Figure** **1**A illustrates. The diameter of LP was 172.1 ± 6.7 nm. After co‐extrusion with normal RBC membrane and apoptotic RBC membrane, biomimetic liposomes were obtained with the size of 253.9 ± 14.6 nm (RLP) and 315.5 ± 15.7 nm (Effero‐RLP) respectively. PDI of each sample was less than 0.3, indicating the homogeneity of particles. TEM images displayed that LP, RLP and Effero‐RLP particles were spherical with hollow structures (Figure 1B; Figure S3, Supporting Information). The prepared LP exhibited neutral surface charge. With cell membrane hybrid, the surface charge of RLP and Effero‐RLP were negative as Figure 1D showed. The encapsulation efficiency of ROSI in LP, RLP and Effero‐RLP were 89.6%, 91.6%, and 86.3% respectively. The drug release was measured in Figure 1E, where ROSI exhibited gradual release trend in biomimetic particles within 48 h. About 30% ROSI of LP was released into medium at 1 h and then dissolved continuously, until reached full release at 24 h. While drug release of RLP and Effero‐RLP were relatively slower. In the first hour, there were just 20% ROSI released from RLP and 14% ROSI released from Effero‐RLP. Until 8 h, about a half of ROSI was released from RLP and Effero‐RLP. Finally, ROSI in RLP and Effero‐RLP were gradually dissolved in medium within 48 h. Moreover, the particle size of liposome was stable within 7 days, while the size of RLP and Effero‐RLP slightly increased as Figure 1F shows. Long‐term stability (30 days) of RLP and Effero‐RLP were also satisfactory (Figure S4, Supporting Information). In addition, drug content in all particles was kept stable after storage, which indicated the satisfied stability for one month of storage.

To observe whether RBC membranes were successfully hybrid with liposomes, LPs were labeled with DiD and cell membrane were labeled with DiO for confocal microscope examination (Figure 1G). These three types of particles were first captured by macrophages to fix them. It was clear that red and green fluorescence were greatly co‐localized, which suggested RBC membrane successfully hybrid with liposome particles. On the other hand, fluorescence intensity of Effero‐RLP was apparently higher than LP and RLP, which suggested that Effero‐RLP with PS exposure improved its uptake by macrophages.

### 2.2 Effero‐RLP Were More Effectively Phagocytosed by Macrophages In Vitro

**Figure 2:**
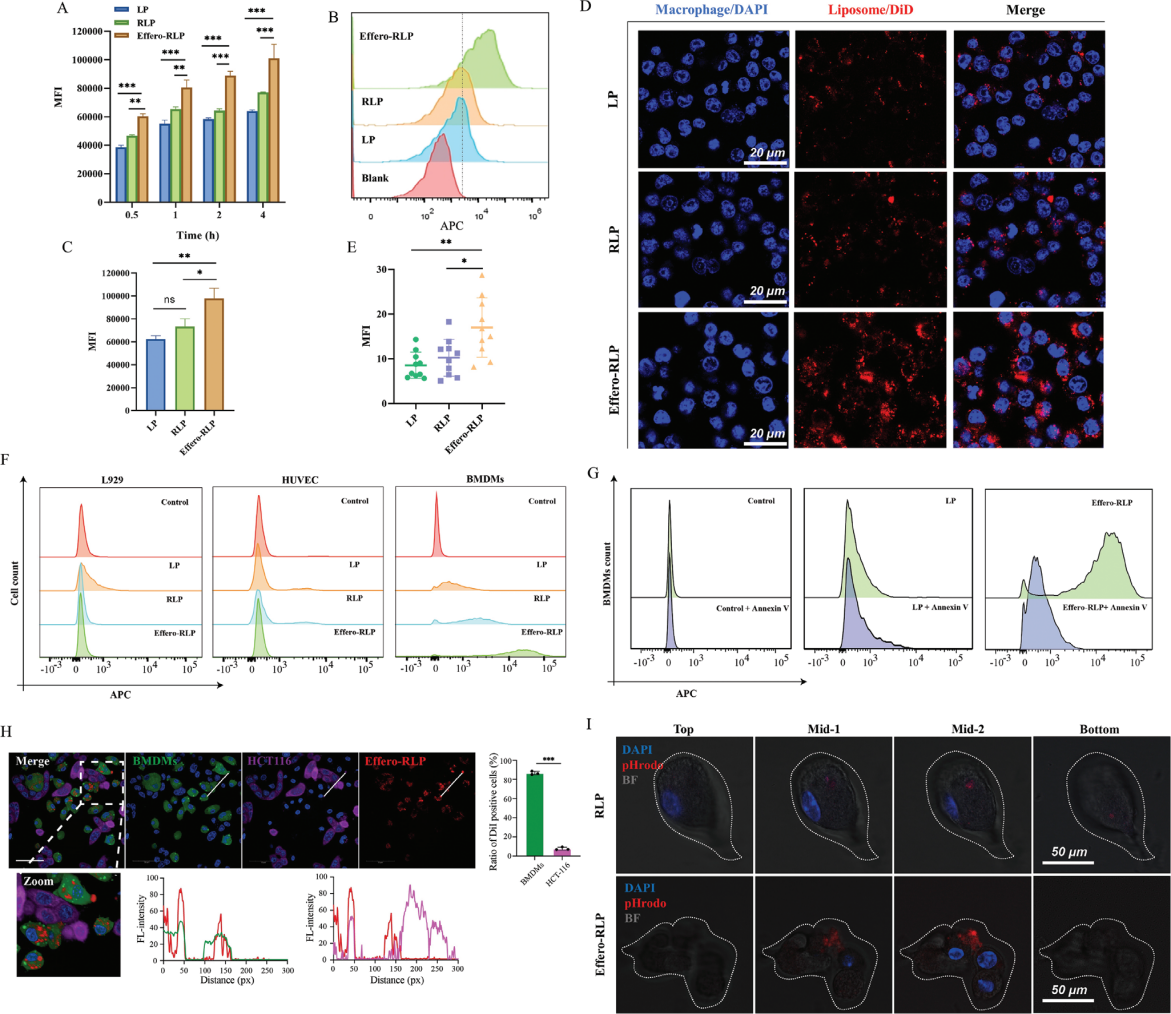
The characterization of macrophage targeting effect of Effero‐RLPs in vitro. A) DiD‐labeled LP, RLP and Effero‐RLP were added into cell culture medium as 20 µg mL^−1^ and incubated with RAW 264.7 for 0.5, 1, 2 and 4 h. The fluorescence intensity of each cell was measured by flow cytometry (n = 3). B) RAW 264.7 were treated by DiD labeled LP, RLP and Effero‐RLP for 2 h, the percentage of DiD positive cells were calculated by flow cytometer. C) After incubating with LP, RLP and Effero‐RLP for 2 h, the fluorescence intensity was analyzed by flow cytometer. D) RAW 264.7 were fixed by PFA, nuclei were stained with DAPI (blue). The uptake of DiD labeled particles by macrophages was observed by confocal microscope. E) 10 cells were randomly selected from confocal images and the fluorescence intensity was quantitatively analyzed by ImageJ. F) L929, HUVEC and BMDM cells were treated with DiD labeled LP, RLP and Effero‐RLP for 2 h and the fluorescence intensity were measured by flow cytometer. G) DiD labeled LP and Effero‐RLP were first pre‐treated with Annexin V and then co‐cultured with BMDM, the fluorescence intensity was measured by flow cytometer. H) The uptake of DiI labeled Effero‐RLP by co‐cultured HCT116 and BMDM cells. I) BMDM were treated with pHrodo labeled RLP and Effero‐RLP, the phagocytosis of these two particles were observed in top, middle and bottom of BMDM by Confocal Laser Scanning Microscope. All significant differences were analyzed by one‐way ANOVA followed by Tukey's honestly significant difference post‐hoc test (ns: no statistical difference, ^*^ *p* < 0.05, ^**^ *p* < 0.01).

To study the cellular uptake of Effero‐RLP by macrophages in vitro, DiD was encapsulated into LP to track particles. RAW 264.7 cells were co‐cultured with LP, RLP and Effero‐RLP for 0.5, 1, 2, and 4 h. It was obvious that with the time increasing, the MFI was gradually higher among these groups (**Figure** **2**A). In contrast, no significant increase of MFI for LP and RLP was observed when cells were incubated with particles for 2 h, indicating the saturated uptake of these particles. Then, RAW 264.7 cells were co‐cultured with DiD labeled particles for 2 h to investigate macrophages uptake for LP, RLP and Effero‐RLP, the percentage of DiD positive cells were analyzed in Figure 2B. There were 19.8% positive cells in LP group, while 21.1% positive cells for RLP and 53.7% positive cells for Effero‐RLP, which displayed Effero‐RLPs were more efficiently uptake by RAW 264.7 cells. Compared with LP and RLP, the MFI of Effero‐RLP in Figure 2C was markedly increased (p < 0.05). This observation was confirmed by confocal microscopy as Figure 2D,E showed.

Even though the enhanced macrophages uptake of Effero‐RLP was demonstrated, whether this capacity was specific for macrophages remained to be claimed. Mouse fibroblast cell line L929, human umbilical vein endothelia cells (HUVEC) and Bone Marrow Derived Macrophages (BMDM) were chosen as typical cell types of fibroblasts (with certain phagocytic ability), vascular cells and macrophages to perform uptake experiment. By treating them with DiD labeled samples for 2 h, the fluorescence intensity in each cell line was measured in Figure 2F. In L929 cell line, the uptake of LP was slightly increased, but there was almost no uptake for RLP and Effero‐RLP. HUVEC cell existed non‐uptake for three liposome particles. When treated with BMDM, the uptake of Effero‐RLP was significantly higher than LP and RLP consistent with RAW 264.7 uptake. Besides, the enhanced capturing ability of Effero‐RLP in BMDM was reversed by Annexin V, a PS blocker, while Annexin V displayed no effect on LP uptake in Figure 2G. It provided the evidence that the macrophage targeting effect of Effero‐RLP relied on the PS recognition by macrophages. In addition, when HCT116 and BMDM cells were co‐cultured in same cell plate and incubated with DiI labeled Effero‐RLPs, most of particles were phagocytosed by macrophages that DiI signal was well co‐localized with BMDM. But almost no DiI fluorescence co‐localized with HCT116 cells as Figure 2H displayed, which indicated the specific macrophage targeting of Effero‐RLP.

To further confirm whether Effero‐RLPs could be internalized by macrophages rather than being attached on the surface of macrophages, the pH‐sensitive dye pHrodo was used for labeling RLP and Effero‐RLP. Due to the endpoint of macrophage phagocytosis was the fusion of phagosome and lysosome, in which pHrodo was activated in acidic internal environment that burst red fluorescence. As Figure 2I showed, red fluorescence in Effero‐RLP was viewed obviously in the middle of BMDM without overlapping with blue fluorescence in nucleus, while no red signal was observed on the top and bottom of BMDM. For RLP, red fluorescence was also absent on the top and bottom of BMDM, whereas slight red fluorescence appeared in middle of BMDM. Indeed, Effero‐RLP could be internalized into the macrophages instead of just attaching on the macrophages' surface. Only a small amount of RLP was phagocytosed by macrophages consistent with the above observation in flow cytometry results.

### 2.3 The In Vivo Distribution of Effero‐RLP on Colitis Mouse

**Figure 3:**
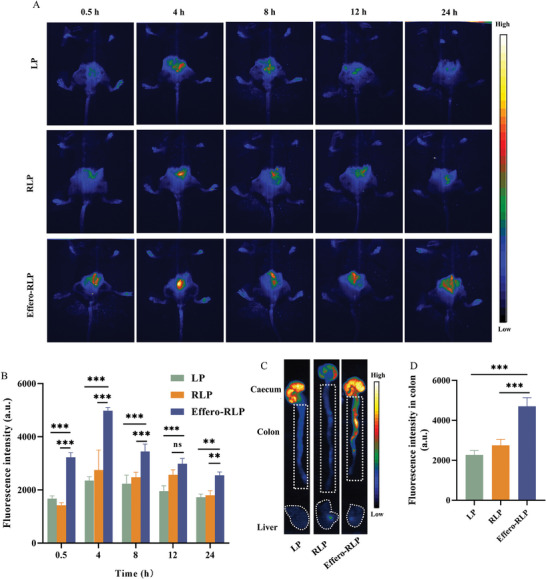
The bio‐distribution of LP, RLP and Effero‐RLP on colitis mouse. A) Biodistribution of DiD labeled LP, RLP and Effero‐RLP on colitis mouse after injection for 24 h; B) Quantitative analysis of fluorescence intensity in lower abdomen for each mouse at different time points; C) The fluorescence distribution of DiD‐labeled particles in colons after 24 h of injection; D) The fluorescence intensity in colons for each treatment group. Data are shown as mean ± SD, n = 3. Significance analyzed by one‐way ANOVA followed by Tukey's honestly significant difference post‐hoc test (^***^ *p* < 0.001).

To further validate the in vivo distribution of liposome particles, DiD labeled LP, RLP and Effero‐RLP were injected into Dextran Sulfate Sodium Salt (DSS)‐induced colitis mice to observe bio‐distribution. After injection for 0.5, 4, 8, 12 and 24 h, each mouse was anesthetized by isopropanol and whole body was imaged at λex = 644 nm as **Figure** **3**A and Figure S5 (Supporting Information) showed. For LP treatment group, most of LP accumulated in intestine after injection for 4 h, then the fluorescence signal decreased in 8 h. After 12 h, DiD fluorescence intensity exhibited a significant decline throughout the entire mouse body, eventually reaching near disappearance within 24 h. In RLP treatment group, particles accumulated in intestine with highest fluorescence intensity after 4 h and gradually declined until 24 h. When Effero‐RLP injected, particles quickly accumulated in intestinal sites after 0.5 h and reached the maximum at 4 h. The fluorescence intensity exhibited a decreasing trend after 4 h, however, it remained at a consistently higher level for the subsequent 20 h. To quantify the accumulation of different particles in intestinal site, the fluorescence intensity of each mouse at different time points was recorded simultaneously in Figure 3B. The accumulation of all three particles in the intestine reached a maximum of 4 h after injection, followed by a subsequent decline. It was evident that Effero‐RLP exhibited significantly higher fluorescence intensity compared to LP and RLP from 0.5 to 24 h, especially Effero‐RLP still maintained at a relatively higher level in 24 h. To confirm whether particles accumulated in colon tissue, mice were sacrificed with high‐concentration CO_2_ and colon tissues were collected. The fluorescence distribution was imaged in Figure 3C, and the fluorescence intensity was quantitatively analyzed in Figure 3D. The DiD intensity of Effero‐RLP in ex vivo colon tissue was highest among groups and there was almost no fluorescence in other two groups, which was consistent with the MFI in abdominal of whole mouse body. Due to the great macrophage targeting property of Effero‐RLP, biomimetic liposomes could be phagocytosed by macrophages throughout the body including kupffer cells in liver. Effero‐RLP achieved high‐efficiency intestine localization for two reasons: 1) Intestine harbored the largest portion of resident macrophages in the body;^[^ ^21^ ^]^ 2) Intestinal inflammation in colitis models recruited a large number of circulating macrophages to the site of inflammation.^[^ ^22^ ^]^ Therefore, Effero‐RLP could effectively target macrophages and accumulate in inflammatory colon tissue for IBD treatment with satisfied drug delivery efficiency.

### 2.4 The Effero‐RLP‐ROSI Displayed Superior Anti‐Inflammatory Activity In Vitro

**Figure 4:**
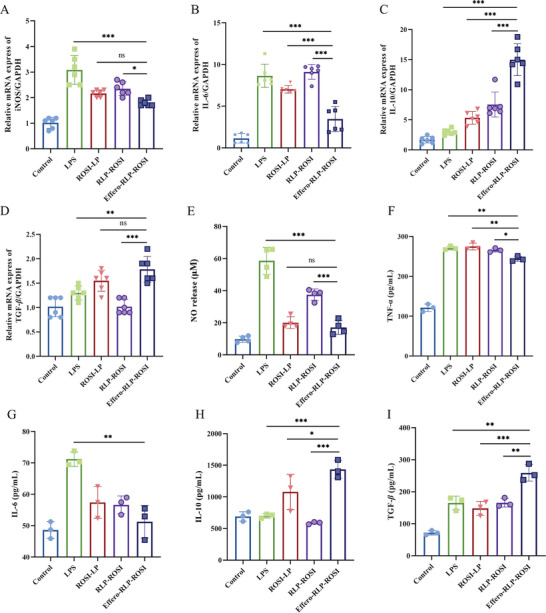
Anti‐inflammatory response of Effero‐RLP‐ROSI on RAW 264.7. The mRNA expression of iNOS A), IL‐6 B), IL‐10 C) and TGF‐*β* D) were measured by real‐time PCR. The NO concentration released in cell culture medium was detected by NO assay kit E); while the release of cytokines including TNF‐*α* F), IL‐6 G), IL‐10 H) and TGF‐*β* I) in supernatant of macrophages were determined by ELISA kits. Data were Mean  ±  SD, one‐way ANOVA followed by Tukey's honestly significant difference post‐hoc test was used for significance analysis (^*^ *p*  <  0.05; ^**^ *p* < 0.01; ^***^ *p* < 0.001, ^****^ *p* < 0.0001).

The liposome particles were utilized to encapsulate ROSI, an agonist for peroxisome proliferators‐activated receptor γ (PPAR‐γ), to enhance the anti‐inflammatory effect of Effero‐RLP, which was known for its superior macrophages and inflammation targeting ability. ROSI liposomes (ROSI‐LP) and normal RBC membrane hybrid liposomes (RLP‐ROSI) were also prepared as comparison groups to compare differences. First, RAW 264.7 cells were pre‐treated with LPS (500 ng mL^−1^) for 24 h, while other RAW 264.7 without LPS treatment were cultured as a control group. After 10 µM of three liposomes treatment for 12 h, the mRNA expression of cytokines in each treatment group was measured by real‐time PCR in **Figure** **4**A–D. RAW 264.7 cells were stimulated by LPS with the increased expression of iNOS and IL‐6. Compared to ROSI‐LP and RLP‐ROSI treatment groups, Effero‐RLP‐ROSI effectively attenuated the intracellular expression of iNOS and IL‐6. For anti‐inflammatory cytokines, Effero‐RLP‐ROSI significantly increased the expression of IL‐10 and TGF‐*β*. Accordingly, the concentrations of NO and cytokines released in cell supernatant were measured by ELISA kits as Figure 4E–I showed. Upon LPS stimulation, the substantial release of NO and pro‐inflammatory cytokines including TNF‐*α* and IL‐6 were observed in cellular medium at high level. After liposome treatment, the release of NO was attenuated to varying degrees, with Effero‐RLP‐ROSI exhibiting the most pronounced inhibitory effect. The liposomes mentioned above demonstrated down‐regulation effect on pro‐inflammatory cytokines, including TNF‐*α* and IL‐6. Impressively, Effero‐RLP‐ROSI had a statistically significant impact compared to others, resulting in the lowest concentration of TNF‐*α* and IL‐6. In contrast, Effero‐RLP‐ROSI improved the release of IL‐10 and TGF‐*β*, which were associated with efferocytosis and possess anti‐inflammation properties. The cellular study provided strong evidence supporting the potent inhibitory effects of Effero‐RLP‐ROSI on pro‐inflammatory factors and its ability to enhance anti‐inflammatory responses with superior efficacy.

### 2.5 The Effero‐RLP‐ROSI Displayed Superior Therapeutic Efficacy on Colitis Mouse Model

**Figure 5:**
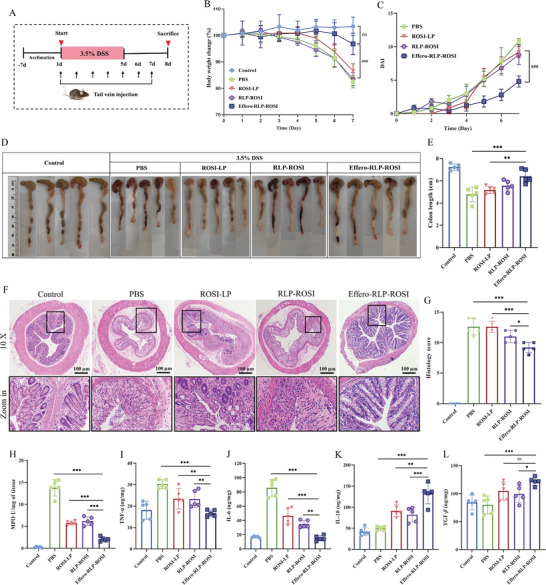
Therapeutic effect of Effero‐RLP‐ROSI on colitis mice mouse. A) A schematic diagram of the treatment procedure for DSS‐induced colitis mice model. C57BL/6 mice were fed with water or 3.5% DSS water for 5 days to establish control and colitis mouse model. Meanwhile, PBS, ROSI‐LP, RLP‐ROSI or Effero‐RLP‐ROSI were injected into mice by tail vein once a day for 7 days. Then mice were sacrificed at 8th day for further analysis. B) The body weight change of each mouse in different groups. C) The DAI score of each mouse in different treatment groups. D) The photographs of colons harvested from mice in different treatment groups and E) the corresponding quantified lengths. F) The hematoxylin‐eosin (H&E) staining images of colons tissues in different treatment groups. G) The colonic histological damage score for HE staining images evaluation. H) MPO level in colon tissue. The expression level of TNF‐*α* I), IL‐6 J), IL‐10 K) and TGF‐*β* L) cytokines in colon tissue were measured by ELISA kits. Data were represented as mean ± SD, all significant differences were determined by one‐way ANOVA followed by Tukey's honestly significant difference post‐hoc test (ns: no statistically difference, ^*^ *p*  < 0.05, ^**^ *p*  < 0.01, ^***^ *p * < 0.001; # represented that compared with Effero‐RLP‐ROSI group, ^##^ *p*  < 0.01, ^###^ *p*  < 0.001).

Next, anti‐inflammation effects of different liposome formulations were further investigated in DSS‐induced colitis mice model. The schematic demonstration of the establishment of colitis mice model is depicted in **Figure** **5**A. Within 5 days of DSS administration, mice with PBS treatment showed serious colitis symptoms, including obvious body weight loss, colon shortening and increasing disease activity index (DAI). Under the administration of various liposome formulations at 0.5 mg kg^−1^, the symptoms of intestinal inflammation were alleviated to varying degrees. The mice in the PBS, ROSI‐LP, and RLP‐ROSI treatment groups exhibited significant weight loss over a period of 7 days, whereas the Effero‐RLP‐ROSI group displayed comparatively milder reduction in body weight (Figure 5B). On the other hand, except Effero‐RLP‐ROSI, other treatment groups exhibited the gradual increase of DAI with serious loose stool and visible blood around anus from 4th to 7th day. Mice with Effero‐RLP‐ROSI treatment appeared slightly loose stool and hemoccult positive symptoms on 7th day (Figure 5C). As shown in Figure 5D,E, the colon length was observed to decrease in the PBS, ROSI‐LP, and RLP‐ROSI treatment groups corresponding to increasing inflammation severity; however, only the Effero‐RLP‐ROSI group exhibited a slight reduction in colon length comparable to healthy group. In terms of IBD symptoms, Effero‐RLP‐ROSI exhibited the most obvious maintenance of body weight, DAI, and colon length from all treatment groups. Then, colon tissue damage of each mouse was assessed by HE staining, which reflected the histological damage including crypt misstructure, inflammatory cells infiltration and mucosal disruption. Compared to control group, The epithelia and crypt exhibited evident damages, accompanied by a substantial infiltration of inflammatory cells in the colon tissues induced by DSS (Figure 5F,G). Effero‐RLP‐ROSI was effectively capable of repairing tissue damage, evidenced by maintaining the integrity of epithelial mucus, restoring crypt, and reducing the infiltration of inflammatory cells. While other liposome formulations exhibited the limited alleviation of inflammation in colon tissue morphology as some visible damages still persisted. Furthermore, MPO served as a peroxidase enzyme indicative of neutrophil infiltration at the site of inflammation. The expression of enzyme and inflammatory cytokines in colons of mice exhibited variations throughout the progression of IBD. Upon treatment with different liposome formulations, the expression level of MPO and inflammatory cytokines in lysis solution of colon tissue were also analyzed. The MPO level was significantly elevated in the colitis mice model, indicating a substantial infiltration of neutrophils in the colon. With the treatment of above three liposomes, the expression of MPO was downregulated. Remarkably, Effero‐RLP‐ROSI exhibited the most pronounced effects, leading to a dramatic reduction in MPO level (Figure 5H). Then the release of pro‐inflammatory cytokines like TNF‐α and IL‐6 and anti‐inflammatory cytokines like IL‐10 and TGF‐β in colon tissue homogenate were measured by ELISA assay. As Figure 5I–L showed, the protein level of TNF‐*α* and IL‐6 exhibited a significant increase concomitant with the progression of IBD. Compared with other treatment groups, the secretion of these pro‐inflammatory cytokines was effectively inhibited by Effero‐RLP‐ROSI. The protein level of anti‐inflammatory cytokines, including IL‐10 and TGF‐β, were significantly increased by Effero‐RLP‐ROSI with superior efficacy. IL‐10 was the well‐recognized anti‐inflammatory cytokine that responded to suppressing pro‐inflammatory cytokines secretion. TGF‐β was the multifunctional cytokine that regulated inflammatory process, particularly in intestinal sites.^[^ ^23^ ^]^ The release of IL‐10 and TGF‐β can be triggered by efferocytosis to maintain the tissue homeostasis.^[^ ^24^ ^]^ The remarkable up‐regulation of IL‐10 and TGF‐β in Effero‐RLP‐ROSI group further validated the phagocytosis of Effero‐RLP by macrophages via the efferocytosis machinery. Moreover, the biomimetic liposome carriers without ROSI and free ROSI were also administrated to colitis mice to compare the treatment effect with Effero‐RLP‐ROSI in Figure S6 (Supporting Information). It was obvious that apoptotic cell membrane hybrid liposome carrier alone did not exhibit any therapeutic effects on colitis. Compared with free ROSI, Effero‐RLP‐ROSI showed the improved anti‐inflammatory effect. Therefore, considering the body weight, DAI, colon length, histology of colon and the level of inflammatory cytokines, Effero‐RLP‐ROSI demonstrated superior therapeutic efficacy in DSS‐induced colitis mice model.

### 2.6 Effero‐RLP‐ROSI Promoted the Reprograming of Pro‐Inflammatory Macrophages into Anti‐Inflammatory Phenotypes in Colon Tissue of Colitis Mouse

**Figure 6:**
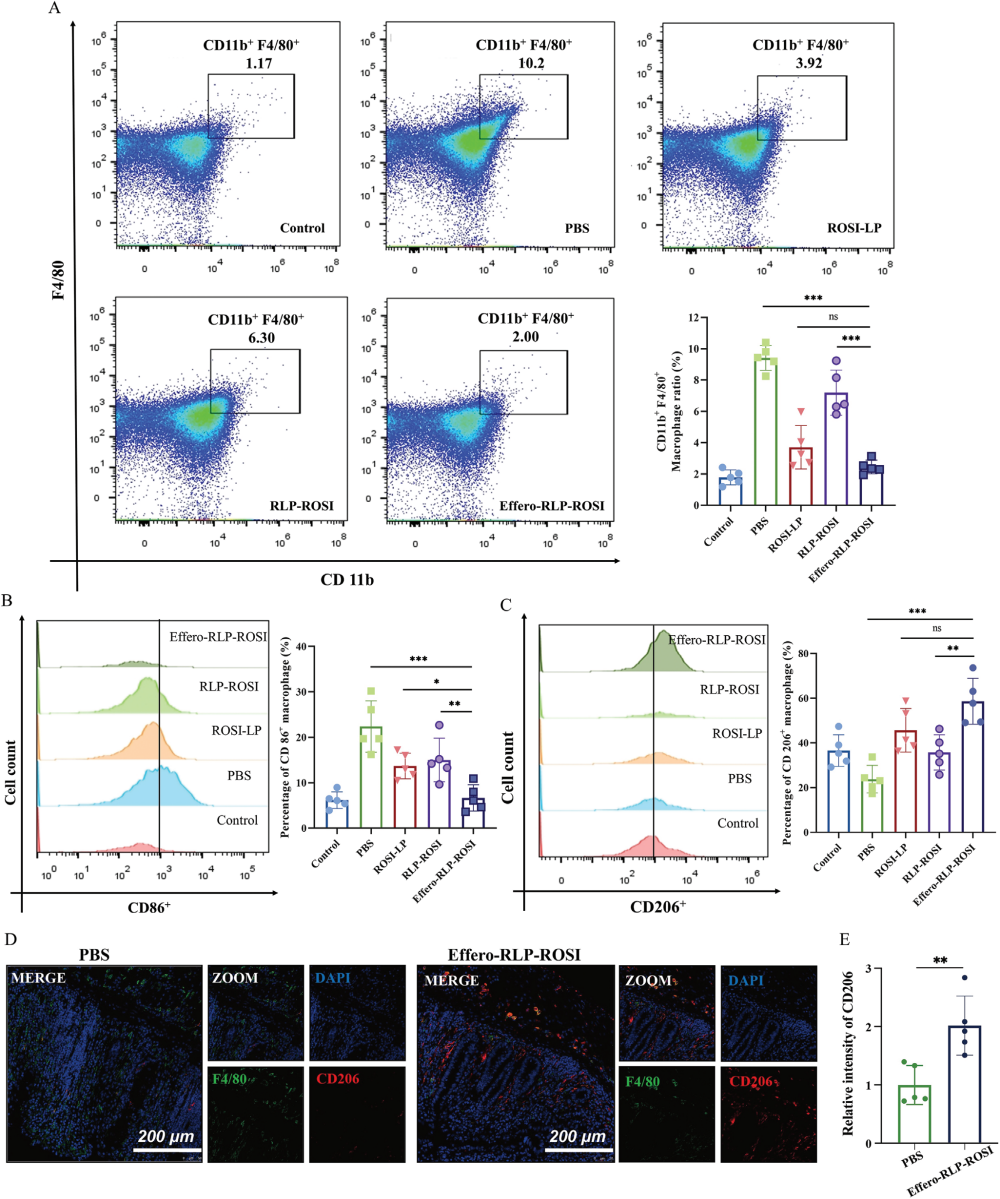
The analysis of macrophage polarization in colon tissue. A) The percentage of CD11b+ F4/80+ macrophages from colonic lamina propria in different treatment group; B) The percentage of CD86 positive macrophages in colon tissue; C) The percentage of CD206 positive macrophages in colon tissue; D) The immunofluorescence staining images of CD206 of colons with PBS and Effero‐RLP‐ROSI treatment; E) The corresponding fluorescence intensity of CD206 of colons with PBS and Effero‐RLP‐ROSI treatment. Data are shown as mean ± S.D. (n = 5). ns: no statistically difference; ^*^p < 0.05; ^**^p < 0.01; ^***^p < 0.001.

To investigate macrophage regulatory functions of Effero‐RLP‐ROSI in colitis mice models, the polarization of macrophages in the colon tissue was determined. First, the population of CD11b^+^ F4/80^+^ macrophages among all kinds of cells in colonic lamina propria was analyzed by flow cytometry in **Figure** **6**A. Unlike healthy mice, increasing number of macrophages with percentage of 10% were infiltrated in inflammatory colon of colitis mice. As expected, with the treatment of Effero‐RLP‐ROSI, the number of macrophages in colon was prominently reduced, whereas that was reduced in other treatment groups to a limited degree. As ROSI has been reported to modulate macrophage polarization with anti‐inflammatory effects, how Effero‐RLP‐ROSI affected macrophage polarization was further studied in Figure 6B,C. In colon tissue of colitis mice, there was an upregulation of pro‐inflammatory M1 type CD86^+^ macrophages, while a downregulation was observed in anti‐inflammatory M2 type CD206^+^ macrophages. Following administration of various liposomes, there was a decrease in CD86^+^ macrophages and an increase in CD206^+^ macrophages. The lower expression of CD86^+^ and the higher expression of CD206^+^ macrophages in Effero‐RLP‐ROSI treatment group indicated the effective regulation of macrophages from pro‐inflammatory phenotype to anti‐inflammatory phenotype. Consistent with the macrophages polarization results measured by flow cytometry, immunofluorescence staining of colon tissue in Effero‐RLP‐ROSI treatment group showed significant increase in CD206 fluorescence intensity (Figure 6D), indicating an augmentation of anti‐inflammatory macrophage phenotypes. Collectively, it can be concluded that Effero‐RLP‐ROSI has multifunctional properties with potent anti‐inflammation effects by the modulation of macrophage polarization.

**Figure 7:**
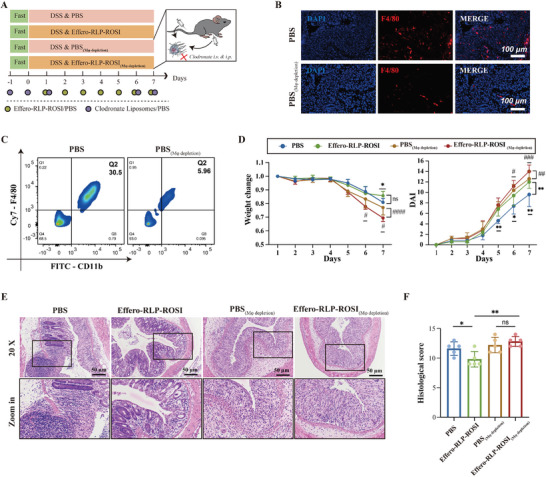
Macrophages depletion abolished therapeutic effect of Effero‐RLP‐ROSI on colitis mouse. A) Schematic diagram of the treatment procedure for macrophages depletion and DSS‐induced colitis mice model. C57BL/6 mice were fed with water or 3.5% DSS water for 5 days to induce IBD symptoms. Clodronate was injected on the first three days and on the sixth, seventh day. Meanwhile PBS and Effero‐RLP‐ROSI were injected into mice by tail vein for 7 days. Then mice were sacrificed at 8th day for further analysis; B) The number of macrophages in mouse liver was analyzed by immunofluorescence staining. C) After administration of clodronate, the macrophages in mouse peritoneal cavity were measured by flow cytometer. D) With the treatment of PBS and Effero‐RLP‐ROSI, the body weight and DAI in DSS treated mouse with or without macrophages depletion were analyzed. E) Representative HE staining images of colon tissues in PBS and Effero‐RLP‐ROSI treatment groups. F) Corresponding histological score for each treatment group. Data are shown as mean ± S.D. (n = 5). ns: no statistically difference; ^*^p < 0.05; ^**^p < 0.01; ^#^ represented that compared with Effero‐RLP‐ROSI_(Mφ depletion)_ group, ^#^p < 0.05; ^##^p < 0.01, ^###^p < 0.001.

Then the macrophages depletion (Mφ depletion) mouse model was established to confirm the therapeutic effect of Effero‐RLP‐ROSI relying on macrophages. As **Figure** **7**A described, clodronate was encapsulated into liposome for macrophages depletion by both i.v. and i.p. on first three days and last two days. Then the number of macrophages in mouse colon and peritoneal cavity was analyzed to validate macrophages depletion efficacy. As immunofluorescence staining images of colon tissues displayed (Figure 7B), the fluorescence intensity of F4/80 in clodronate treatment group was significantly weakened and even disappeared in some regions, suggesting the colonic macrophages were effectively depleted. Then macrophages in mouse peritoneal cavity were also collected and analyzed by flow cytometer in Figure 7C. It was clear that the percentage of macrophages was decreased from ≈30% to 6% after clodronate administration, which indicated the successful trial for macrophage depletion.

Normal mice and macrophages depleted mice were fed with 3.5% DSS water for 5 days to induce colitis model. PBS and Effero‐RLP‐ROSI were administrated to mice individually by intravenous injection. In terms of body weight of mouse, with the treatment of Effero‐RLP‐ROSI, the weight loss of mouse was effectively inhibited. But when macrophages depleted, PBS and Effero‐RLP‐ROSI treatment group experienced more weight loss, especially for Effero‐RLP‐ROSI. DAI during treatment period displayed similar trend with body weight change in Figure 7D. Effero‐RLP‐ROSI could effectively alleviate symptoms of colitis with the presence of macrophages, whereas the symptoms were getting worse in macrophages depleted mice. Similarly, histological analysis showed that Effero‐RLP‐ROSI effectively alleviated the colonic crypts disruption and inhibited inflammatory cell infiltration, such alleviating effect was also abolished in macrophages depleted mice (Figure 7E,F). Therefore, the multifunction of Effero‐RLP‐ROSI indeed relied on macrophages to exert anti‐inflammatory effect.

**Figure 8:**
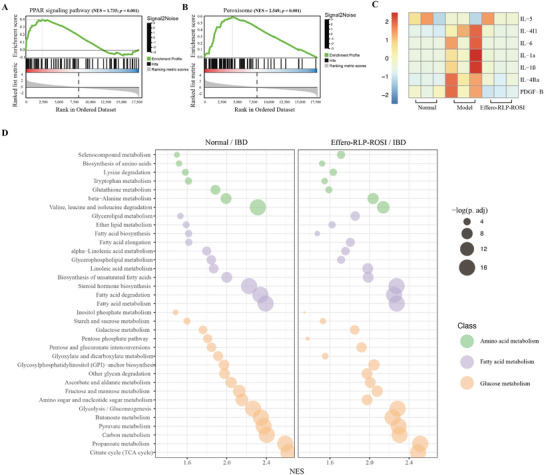
RNA‐seq analysis revealed the interaction mechanisms and biological pathways of the Effero‐RLP on colitis mouse. A) and B) Gene Set Enrichment Analysis (GSEA) of enriched signaling pathways of Effero‐RLP treated group compared with Model group. C) Expression pattern of PPAR‐γ associated inflammatory cytokines across normal group, PBS treated group, and Effero‐RLP‐ROSI treated group. D) Metabolism pathways alteration of normal group and Effero‐RLP‐ROSI treated group compared with IBD group. Data are visualized by using R software (v4.3.1) (n = 3). Normalized enrichment score (NES). NES > 0 upregulated than model group; NES < 0 downregulated than model group.

To better understand the interaction mechanisms and biological pathways of the Effero‐RLP, the RNA sequencing (RNA‐seq) was performed. Because ROSI as a specific agonist of PPAR‐γ, can lead to the activation of peroxisomal biosynthesis, the relevant pathways activated by Effero‐RLP‐ROSI treatment were first checked. The Gene Set Enrichment Analysis (GSEA) of RNA‐seq data facilitated the comprehension of distinct expression patterns in specific biological pathways. Based on such analysis, it could be found that both PPAR signaling pathway (NES = 1.735) and peroxisome pathway (NES = 2.549) were significantly upregulated by Effero‐RLP‐ROSI treatment compared with PBS treated group, indicating a direct activation of PPAR‐γ by Effero‐RLP‐ROSI (**Figure** **8**A,B). PPAR‐γ was demonstrated to downregulate pro‐inflammatory cytokines production such as IL‐4, IL‐5, and IL‐6, but also to interfere with profibrotic molecules such as Platelet‐Derived Growth Factor (PDGF), IL‐1 and TGF‐*β*.^[^ ^25^, ^26^ ^]^ To further confirm the anti‐inflammatory mechanism of Effero‐RLP‐ROSI, the expression pattern of the above mentioned PPAR‐γ associated inflammatory cytokines across normal mice, colitis mice (PBS treated), and Effero‐RLP‐ROSI treated mice were profiled. It demonstrated that Effero‐RLP‐ROSI could regulate these cytokines expression from disease state to healthy state (Figure 8C). Furthermore, PPAR signaling pathway played a vital role in regulating organism metabolism including not only fatty acid metabolism but also glucose metabolism and amino acid metabolism.^[^ ^27^, ^28^, ^29^ ^]^ Then whether these above metabolism pathways were also regulated by Effero‐RLP‐ROSI was checked by GSEA analysis. Interestingly, the expression pattern of signaling pathways in fatty acid metabolism, glucose metabolism and amino acid metabolism between healthy group and Effero‐RLP‐ROSI treated group exhibited very strong consistency compared with the PBS treated group (Figure 8D), indicating Effero‐RLP‐ROSI could attenuate the colitis induced metabolism dysregulation and maintain the tissue homeostasis.

## 3 Discussion

For inflammatory disease treatment, the complicated physiological environment restricted the therapeutic efficiency in clinic and how to accurately deliver drugs into inflammatory site was still a challenge. It was well known that macrophages were critical for the initiation, maintenance, and resolution of inflammation. The functions of macrophages including antigen presentation, phagocytosis and immunomodulation were response for the development of inflammation. Therefore, regulating macrophage function was an effective way to alleviate inflammation. Excitingly, efferocytosis mediated natural recognition between Effero‐RLP and macrophages dramatically improved macrophage targeting efficiency, meanwhile promoting particle accumulation at inflammatory site. ROSI was the agonist of PPAR‐γ that could induce macrophages polarization into anti‐inflammatory phenotype.^[^ ^30^ ^]^ Indeed, efferocytosis‐mediated macrophage targeting further enhanced the regulatory effect of ROSI on macrophages and improved anti‐inflammatory effect.

The current clinical therapies for IBD includes aminosalicylate, steroid, immunosuppressive drugs, probiotic, monoclonal antibody, and surgical treatment. However, there were still some risks in unfavorable prognosis and side effects. Currently, nanotechnologies were developed for small molecules and biomedicines delivery to improve therapeutic efficiency. Nanoparticles were designed as appropriate size that could be transferred through inflammatory barrier through enhanced permeability and retention (EPR) effect to deliver particles with passive targeting, but with lower efficiency.^[^ ^31^ ^]^ Some receptors were highly expressed on the surface of macrophages when inflammation occurred, which provided binding sites for active targeting, such as folate, hyaluronic acid and mannose modified nanoparticles to achieve macrophage targeting and improve therapeutic effect.^[^ ^32^, ^33^, ^34^ ^]^ But the binding efficiency was usually limited by the complex in vivo environment, resulting in the challenge in clinical application. In addition, the newly synthesized materials applied for nanoparticles had potential toxicity, which was the concern in novel drug delivery system application. As Figure S7 (Supporting Information) displayed, PS modified liposomes accelerated blood coagulation as PS was reported to activate thrombosis.^[^ ^35^ ^]^ Whereas Effero‐RLP had no accelerating trend, which confirmed the great biocompatibility and safety. As intestine harbored the largest portion of macrophages, this study focused on macrophage targeting and immunomodulatory to achieve precise drug delivery and minimize side effect. In the cases of colitis, tissue resident macrophages in gut were insufficient for maintaining stability of intestinal microenvironment and performing tissue repair.^[^ ^5^ ^]^ More macrophages derived from monocytes were recruited into inflammatory colon sites with the functions of suppressing pro‐inflammatory response, regulating inflammation related cytokines, and repairing damaged tissue, which were essential for tissue immunity and homeostasis.^[^ ^22^, ^36^, ^37^ ^]^ Unlike the classically constructed nanoparticles, biomimetic nanoparticles with higher biocompatibility and great biological properties developed as the hot topic in current research. Many previous studies applied cell membrane for biomimetic nanoparticles preparation, which proved the feasibility and advantages of this strategy. Herein, Effero‐RLP‐ROSI was the efferocytosis mediated biomimetic particles that could be precisely recognized by macrophages and recruited to inflammatory colon sites. It creatively proposed a targeting strategy based on the biological process of efferocytosis, which was a strategy that kills two birds with one stone. Moreover, it was convinced that Effero‐RLP exhibited specific selectivity for macrophages rather than other cells, and particles were indeed phagocytized by macrophages to regulate macrophages' functions. As it has been proved, liposomes could be transported by blood‐circulating myeloid cells resulting in accumulation in inflammatory regions.^[^ ^38^ ^]^ On the one hand, it achieved precise targeting of macrophages leading to the enhancement of regulating macrophages of ROSI, and on the other hand, it induced efferocytosis‐mediated anti‐inflammatory effects. In this study, the distribution of DiD labeled Effero‐RLP in colitis mice confirmed the effective accumulation in inflammatory sites. Additionally, Effero‐RLP‐ROSI affected the polarization of macrophages into anti‐inflammatory macrophages phenotype by binding PPAR‐γ to demonstrate a great immunoregulation ability. Overall, Effero‐RLP exhibited many advantages in alleviating inflammation. It provided a platform for novel drug delivery system design with higher biocompatibility and efficiency. In future application, Effero‐RLP can be prepared by using host's own RBC to achieve personalized drug delivery. The membrane hybrid method is easy‐operated and the prepared Effero‐RLP is stable during storage for one month. It could be applied to many other inflammation diseases treatment in the future.

## 4 Conclusion

This study employed apoptotic RBC membrane with PS exposure to hybridize with liposomes, aiming to achieve efferocytosis‐mediated macrophage targeting for alleviating inflammation. ROSI as the model drug was encapsulated into Effero‐RLP and phagocytosed into macrophages by efferocytosis to regulate macrophages and further promote anti‐inflammatory effects. The enhanced targeting effect and anti‐inflammatory effect of Effero‐RLP‐ROSI were well confirmed in vitro cell model and in vivo colitis mice model without observed toxicity. Hence, apoptotic cell membrane hybrid biomimetic nanoparticle was a promising way for macrophage targeting to enhance anti‐inflammatory activity and minimize the systemic adverse effects of the drug.

## 5 Experimental Section

### Materials

Rosiglitazone, lecithin, cholesterol, 1,2‐distearoyl‐sn‐glycero‐3‐phosphoethanolamine‐N‐[amino(polyethylene glycol)‐2000] (DSPE‐PEG2000) and 1,1′‐Dioctadecyl‐3,3,3′,3′‐Tetramethylindodicarbocyanine (DiD) were supplied by Aladdin (Shanghai, China). LPS from Escherichia coli O111:B4 was purchased from Sigma (Cat. L4391). Annexin V‐FITC apoptosis detection kit, Propidium iodide (PI) and Cell Counting Kit‐8 were supplied by Beyotime (Shanghai, China). Mouse TNF‐*α* (430904), IL‐6 (431304), IL‐10 (431414) and TGF‐*β* (433007) ELISA Kits were purchased from Biolegend (San Diego, CA, USA). The primers for PCR measurement were synthesized by BGI group. Penicillin‐streptomycin, trypsin, fetal bovine serum (FBS), and Dulbecco's modified Eagle's medium (DMEM) were purchased from Gibco (Thermo Fisher Scientific, USA). All other chemical reagents were of analytical or chromatographic grade.

### Preparation of Apoptotic RBCs Cell Membrane

First, the whole blood was collected from C57BL/6 mice and incubated with 2 volumes of Alsever's Solution (composed of 2.05% dextrose, 0.8% sodium citrate, 0.055% citric acid, and 0.42% sodium chloride) at 4 °C. Then the mixture was centrifuged at 1500 rpm for 5 min to remove plasma with three times repeat. The packed RBCs were resuspended with sterile saline solution to prepare 2% volume fraction RBC suspensions and stored at 4 °C.

To induce RBCs apoptosis, 1.25 mM hydrogen peroxide (H_2_O_2_) was added into RBC suspension and co‐incubated for 4 h at 4 °C.^[^ ^39^ ^]^ Subsequently, the apoptosis ratio was measured by Annexin V‐FITC apoptosis detection kit. The resulting apoptotic RBC was washed by PBS and then suspended in a cold hypotonic lysing buffer, comprising of 10 mm Tris‐HCl buffer (pH 7.4), 1 mM CaCl_2_, and 10 µL EDTA‐free protease inhibitor per 1 mL solution. After 4 h, the hemoglobin was removed by centrifugation at 15000 × g for 15 min. The collected RBC ghosts were washed with Tris buffer and stored in PBS. The quantification of collected RBC membrane was performed using BCA protein assay kit. Then RBC membrane was labeled with DiO dye (*λ*_ex_ = 484 nm, *λ*_em_ = 501 nm) at the concentration of 10 µg mL^−1^ through incubating with DiO solution for 20 min.

### Preparation and Characterization of Bionic Liposome Particles

The liposomes were prepared by anti‐solvent method. Lecithin, DSPE‐PEG2000 and cholesterol were first dissolved in 500 µL ethanol at the weight ratio of 3:1:1. ROSI was also added into ethanol at the weight of 1 mg to form homogeneous organic phase. Subsequently, organic phase was injected into 4.5 mL of PBS while stirring at 400 rpm to obtain liposomes (LP). The free drug and residual ethanol were removed by ultrafiltration using ultrafiltration probe (low protein adsorption regenerated cellulose membrane, MWCO 3000 Da, Sigma–Aldrich) at 4000 rpm for 30 min. Additionally, DiD (*λ*_ex_ = 644 nm, *λ*_em_ = 663 nm) was added into lipid solution at 10 µg mL^−1^ to label liposome particles. Efferocytosis‐based biomimetic liposomes (Effero‐RLP) were produced by co‐extruding liposomes and apoptotic RBC membrane at the weight ratio of 10:1 through a 400 nm polycarbonate porous membrane using Anvati's mini‐extruder for 20 cycles. To compare the difference between normal and apoptotic RBC membrane, fresh RBC membrane was also collected and co‐extruded with liposomes using same ways to prepare RBC membrane coated liposomes (RLP). The size and zeta potential of particles were measured by dynamic light scattering (DLS) (Malvern, Worcestershire, WR, UK). The structure of each sample was observed by transmission electron microscopy (TEM), wherein the samples were dropped into copper mesh and negatively stained by phosphotungstic acid under accelerating voltage. The concentration of ROSI that coated into liposomes was measured by high‐performance liquid chromatography (Agilent 1200 series HPLC) with C18 column (Agilent Zorbax; 4.6 × 250 mm, 5 µm). HPLC was conducted with mobile phase composed of sodium acetate (acetic acid to adjust pH to 6)‐methanol (25:75) at the 1 mL min^−1^ flow rate. The encapsulation efficiency (EE) was calculated by the following formula:

(1) $$\mathrm{EE}\;=\frac{\mathrm{Weight}\;\mathrm{of}\;\mathrm{drug}\;\mathrm{in}\;\mathrm{liposome}}{\mathrm{Weight}\;\mathrm{of}\;\mathrm{total}\;\mathrm{drug}}\;\times 100\%$$

Then LP, RLP and Effero‐RLP were stored at 4 °C for 7 days to one month and the particle size was measured to evaluate particle stability. The in vitro release rate of ROSI was investigated by dialysis. First, 1 mL of LP, RLP and Effero‐RLP was transferred into dialysis bag (MWCO 3500 Da) individually and placed into 50 mL medium composed of PBS (pH 7.4) with 1% Tween 80 (v/v). Each sample was stirred at 100 rpm and maintained at 37 °C for 48 h. At the prescheduled time point, 1 mL of dissolution medium was collected for analysis, while an equal volume of fresh medium was added to the dialysis bag. The collected samples were filtered through 0.22 µm membrane to remove the undissolved liposome particles, then drug concentration was measured by HPLC to calculate the drug release of ROSI. Each sample underwent triplicate drug release measurements. To assess whether cell membrane hybrid with liposomes, RAW 264.7 cells were initially incubated with biomimetic liposomes that RBC cell membranes labeled by DiO and liposomes labeled by DiD, followed by imaging using a confocal microscope (TCS SP8, Leica) to see co‐localization of DiO and DiD fluorescence signals.

### The collection and Culture of Bone Marrow‐Derived Macrophages (BMDM)

The femurs and tibias of a 6‐week‐old male C57BL/6 mouse were extracted and immersed in 75% alcohol for 10 min following euthanasia. Subsequently, the bones were placed in a dish containing 1 mL of PBS after the removal of all muscle tissue. To access the bone marrow from the ends, the epiphyses of both femur and tibia were removed. A syringe needle (1 mL) was then inserted into the bone marrow cavity to rinse it with 10 mL of PBS. The resulting cell suspension was filtered using a 70 µm cell strainer and transferred to a sterile centrifuge tube (50 mL). Cells were collected by centrifugation at 1500 rpm, 4 °C for 5 min. Following addition of red blood cell lysate (1 mL), the mixture was incubated for 10 min before neutralizing lysis with an additional volume of PBS (10 mL). Centrifugation at 1500 rpm, 4 °C for another 5 min was performed, followed by adjustment of cell concentration to reach a density of ≈1 × 10^6^ cells mL^−1^. Finally, cells were seeded into a six‐well plate with each well containing four milliliters of cell suspension. A “half volume” medium change was carried out every other day until collection on day six.

### In Vitro Cell Uptake

The macrophage cell line RAW 264.7 was cultured in DMEM medium supplemented with 10% FBS, 100 U mL^−1^ penicillin, and 100 µg mL^−1^ streptomycin under the humidified environment of 5% CO_2_ at 37 °C. Then cells were inoculated into a 12‐well plate for 24 h and then co‐cultured with DiD‐labeled LP, RLP, and Effero‐RLP individually for 2 h. The uptake of different particles by macrophages was assessed by calculating mean fluorescence intensity (MFI) using flow cytometer (BD LSRFortessa™ Cell Analyzer) with analyzing with FlowJo™ v10.8 software. Additionally, RAW 264.7 cells were fixed and stained with Hoechst 33342 dye to visualize uptake behavior by confocal microscope (TCS SP8, Leica). Image analysis was performed using Leica LAS X software.

### Phagocytosis Analysis

L929, HUVEC, and BMDMs were treated with LP, RLP, and Effero‐RLP (stained with DiD) for 2 h. Subsequently, cells were washed and resuspended in PBS for flow cytometry analysis. To block PS, Effero‐RLP was pretreated with 10% (vol/vol) Annexing V for 30 mins. Following pretreatment step, the Effero‐RLP was incubated with BMDMs for additional 2 h as previously described to observe uptake behavior by flow cytometry. Additionally, Effero‐RLP was stained with pHrodo dyes for 30 mins. After washing and resuspending in PBS, Effero‐RLP was incubated with BMDM for 2 h, followed by gentle washing with PBS three times. To perform confocal imaging, BMDMs were quickly fixed by cold methanol (100%, −20 °C), and stained with Hoechst 33342 (1 µg mL^−1^) for 10 mins. Images were acquired using Leica TCS SP8 Confocal Laser Scanning Microscopy System, and Z‐stack was stetted at 1.8 µm.

### BMDMs and HCT116 Co‐Culturing

After culturing BMDMs in vitro for 7 days, the HCT116 cells were plated in BMDMs culturing plates. The medium for co‐culturing is DMEM (containing 10% FBS and 1% PS) and the mixed cells were cocultured for 12 h. Then, the DiI labeled effero‐RLP was added to culturing plates for 3 h. After 5 times PBS washing, the plates were read by a PE Opera Phenix Plus high content fluorescence imaging system.

### Bio‐Imaging of Effero‐RLP

C57BL/6 mice with 8 weeks old of clean grade were purchased from the University of Macau, fed with standard laboratory chow and drinking water under constant environment conditions (22 °C, 12 h light/dark cycle). To induce colitis model, mice freely drank double‐distilled water containing 3.5% (w/v) Dextran Sulfate Sodium Salt (DSS) for 5 days.^[^ ^40^ ^]^ To validate the in vivo distribution, DiD‐labeled LP, RLP and Effero‐RLP were intravenously injected into mice and imaging by in vivo imaging system at predetermined time points. After 24 h, mice were sacrificed in CO_2_ environment and dissected, the colon from anus to cecum was dissected and imaged. The fluorescence intensity of colon was analyzed by Fiji Image J.

### Anti‐Inflammatory Effect of Effero‐RLP In Vitro

To investigate anti‐inflammatory effect of biomimetic liposomes, conventional liposome encapsulated with ROSI was prepared as comparison group (ROSI‐LP). Additionally, two types of biomimetic liposomes were prepared: normal RBC membrane hybrid liposome (RLP‐ROSI) and apoptotic RBC membrane hybrid liposome (Effero‐RLP‐ROSI). To assess anti‐inflammatory effect of each particle, RAW 264.7 cells were cultured and seeded in 24‐well plate at the density of 2 × 10^5^ cells per well and pre‐treated with LPS (500 ng mL^−1^) for 2 h. Each liposome with the concentration of 10 µm was incubated with cells for 12 h. Then supernatants were collected to analyze inflammatory cytokines by enzyme‐linked immunosorbent assay (ELISA) kits. The expression level of interleukin 6 (IL‐6), IL‐10 and transforming growth factor beta (TGF‐*β*) in each treatment group were measured. NO release in cell supernatants was assessed by NO test kit.

After treatment with different liposomes, RAW 264.7 cells were collected and washed three times with cold PBS. Then mRNA was extracted using Trizol (Thermo Fisher, #15596026) method. Reverse transcription to synthesize cDNA was conducted in accordance with the instructions provided by the reverse transcription kit (Accurate Biotechnology #AG11728). After removing genomic DNA, reverse transcription was conducted at 37 °C for 15 min and 85 °C for 5 s, and then samples were stored at −20 °C until the next step. For standard real‐time quantitative reverse transcription PCR (Real‐Time qRT‐PCR) of cDNA samples, the following primers were used:

TNF‐*α* F: 5′‐GCCCACGTCGTAGCAAACCAC‐3′

TNF‐*α* R: 5′‐GCAGGGGCTCTTGACGGCAG‐3′;

IL‐6 F: 5′‐TACTCGGCAAACCTAGTGCG‐3′

IL‐6 R: 5′‐GTGTCCCAACATTCATATTGTCAGT‐3′;

IL‐10 F: 5′‐TTTGAATTCCCTGGGTGAGAA‐3′

IL‐10 R: 5′‐CTCCACTGCCTTGCTCTTATTTTC‐3′;

TGF‐*β* F: 5′‐CTGAACCAAGGAGACGGAATAC‐3′

TGF‐*β* R: 5′‐GGGCTGATCCCGTTGATTT‐3′;

iNOS F: 5′‐CGGCAAACATGACTTCAGGC‐3′

iNOS R: 5′‐GCACATCAAAGCGGCCATAG‐3′.

Assays were performed using the QuantStudioTM 7 Flex System v1.1 (Biosystems Applied by Life Technologies) according to the kit instructions, with two replicates for each experiment.

### Alleviating Inflammation for Colitis Mouse

For colitis mouse therapy, mice were divided into five groups, including control group of healthy mice and DSS‐induced colitis mice with ROSI‐LP, RLP‐ROSI, Effero‐RLP‐ROSI treatment. First, mice were freely drunk with 3.5% (w/v) DSS water for 5 days followed by normal drinking water for another 2 days to induce colitis. Simultaneously, PBS, ROSI‐LP, RLP‐ROSI and Effero‐RLP‐ROSI were intravenously injected into colitis mice individually at the drug of 0.5 mg kg^−1^ for 7 days. Body weight and disease activity index (DAI) scores were recorded every day. As previously described, DAI score encompassed stool viscosity and rectal bleeding as indicators of disease severity was evaluated.^[^ ^41^ ^]^ On the eighth day, mice were sacrificed with high‐concentration CO_2_ followed by collection and weighing of liver and spleen samples. Colon length was measured and collected for subsequent experiments. All mouse breeding and experimental procedures were approved by the Animal Experiment Ethics Committee of the University of Macau (ethics number: UMARE‐010‐2022).

### RNA Isolation and RNA‐Sequencing

Total RNA from frozen colon tissues were extracted using RNAiso Plus according to the manufacture's protocol. RNAs were detected, sequenced, and analyzed by BGI Genomics (Shenzhen, China).

### Hematoxylin‐Eosin (HE) Staining

For histological analysis, distal colon tissues located 0.5 cm above the anal canal were harvested after administration and fixed with 4% paraformaldehyde (PFA) for 24 h at 4 °C. Then tissues were soaked in a series of ethanol and xylene gradients for dehydration before being embedded in paraffin (Leica, Cat No.39601006). Paraffin tissue sections of 4 µm thickness were prepared and stained with hematoxylin and eosin. Following sealing with neutral resin, the tissue samples were observed using conventional optical microscope. HE scores were assigned based on scoring criteria to evaluate epithelial damage and inflammatory cell infiltration.^[^ ^42^ ^]^

### Myeloperoxidase (MPO) Test

The mouse colon tissue from the 1.5–2.5 cm section above the anal line was collected, washed with ice‐cold PBS. An equivalent weight of tissue was minced and homogenized in hexadecyl trimethyl ammonium bromide (HTAB) buffer using a physical homogenizer at 30 Hz for 10 min to obtain the lysate of colon tissue. The resulting lysate was subjected to centrifugation at 13 400 rpm at 4 °C for 5 min to collect the supernatant. 7 µL of tissue homogenate supernatant and 200 µL of substrate containing o‐dianisidine dihydrochloride (0.167  mg mL^−1^) and hydrogen peroxide (0.0005%) were added to 96‐well plate. The optical density (OD) value at 450 nm was measured every 30 s using the spectrophotometer set at 25 °C following for calculation of MPO activity based on the slope of the OD‐time curve.^[^ ^42^ ^]^

### Cytokines in Colon Tissue

After treatment, colon tissue of each mouse was harvested and homogenized in lysis buffer that contained 50 mM Tris buffer, 150 mM NaCl, 2 mL Triton X‐100 and 1% protease inhibitor using physical homogenizer at 30 Hz for 10 min. The supernatant was collected by centrifugation at 3300 g for 5 min. The concentration of TNF‐*α*, IL‐6, IL‐10 and TGF‐*β* in colon homogenate supernatant were measured by ELISA kits following provided protocol.

### The Polarization of Macrophages in Colon Lumina Propria Cell

To analyze the macrophage population in colon tissue, all cells in lumina propria were isolated and collected as previous reported.^[^ ^43^ ^]^ First, the anatomical colon tissues were rinsed three times with cold PBS and then cut into small pieces. The treated tissues were transferred into Ca/Mg‐free HBSS including 2 mM EDTA and shaken at 37 °C for 10 min repeating three times. Subsequently, these tissues were transferred into RPMI medium supplemented with 20% FBS, 1 mg mL^−1^ collagenase IV and 0.05 mg mL^−1^ DNAase for digestion at 37 °C with shaking at 200 rpm for 30 min. Then digestion was stopped by adding HBSS with 10% FBS. The cells were collected by centrifugation at 1000 rpm for 4 min. Finally, the suspensions were filtered through sterile cell strainers to obtain pure cells. To identify the mononuclear cells and separate out macrophages, cell suspensions were stained with antibodies, including CD11b, F4/80, CD86 and CD206. All samples underwent flow cytometer.

### Immunofluorescent Staining

Macrophages in colon were determined through immunostaining with F4/80 and CD206 antibodies. Frozen tissue sections were fixed with ice‐cold 4% PFA for 10 min at room temperature, followed by permeabilization using PBST (0.3% Triton‐100X in PBS) and blocking with 5% BSA. After staining with F4/80 and CD206, sections were incubated with primary antibodies against CD206 or F4/80, followed by secondary antibodies conjugated to Alexa Fluor 647 or Alex Fluor 488 for macrophage identification. Images were captured by Leica TCS SP8 Confocal Laser Scanning Microscopy System and analyzed using ImageJ software.

### Therapeutic Effect of Effero‐RLP‐ROSI on Macrophages Depleted Colitis Mouse

In the depletion study, 100 µL of clodronate liposome was administered intravenously and intraperitoneally to mice. For verification of macrophages depletion, the mice were sacrificed by CO_2_ treatment and the peritoneum lavage was performed with PBS. Peritoneal macrophages were blocked with 5% BSA and stained with CD11b and F4/80 antibodies. Macrophages were determined as double positive cells by flow cytometry. Then macrophages depleted mice received DSS water to induce IBD symptoms. Effero‐RLP‐ROSI (0.5 mg kg^−1^) was intravenously injected into mice. The body weight and DAI were recorded, while colon tissue damage was evaluated by HE staining.^[^ ^42^ ^]^

### Statistical Analysis

Statistical analysis was performed using Microsoft Excel and Prism 7.0 (GraphPad). Student's t‐test or one‐way analysis of variance (ANOVA) was performed respectively. One‐way repeated measures ANOVA was used to compare differences in scores over time. Each Data was presented as mean ± SD, and differences were considered significant if *p* < 0.05 (^*^ *p* < 0.05, ^**^ *p* < 0.01, ^***^ *p* < 0.001, ^****^ *p* < 0.0001, unless otherwise stated).

## Conflict of Interest

The authors declare no conflict of interest.

## Supporting information

Supporting Information

## Data Availability

The data that support the findings of this study are available from the corresponding author upon reasonable request.
