# Correction to [Synthetic Biomimetic Liposomes Harness Efferocytosis Machinery for Highly Efficient Macrophages‐Targeted Drug Delivery to Alleviate Inflammation]

**DOI:** 10.1002/advs.202515155

**Published:** 2025-08-22

Han R, Ren Z, Wang Q, Zha H, Wang E, Wu M, Zheng Y, Lu JH. Synthetic Biomimetic Liposomes Harness Efferocytosis Machinery for Highly Efficient Macrophages‐Targeted Drug Delivery to Alleviate Inflammation. Adv Sci (Weinh). 2024 Aug;11(29):e2308325.

We identified an inadvertent error in Figure 5D on page 2308325 (page 8 of 16). Specifically, in the control group and the RLP‐ROSI group, one colon tissue image in each group was mistakenly shown two times (the second and fifth images in the control group, and the third and fifth images in the RLP‐ROSI group) (Supporting Information).


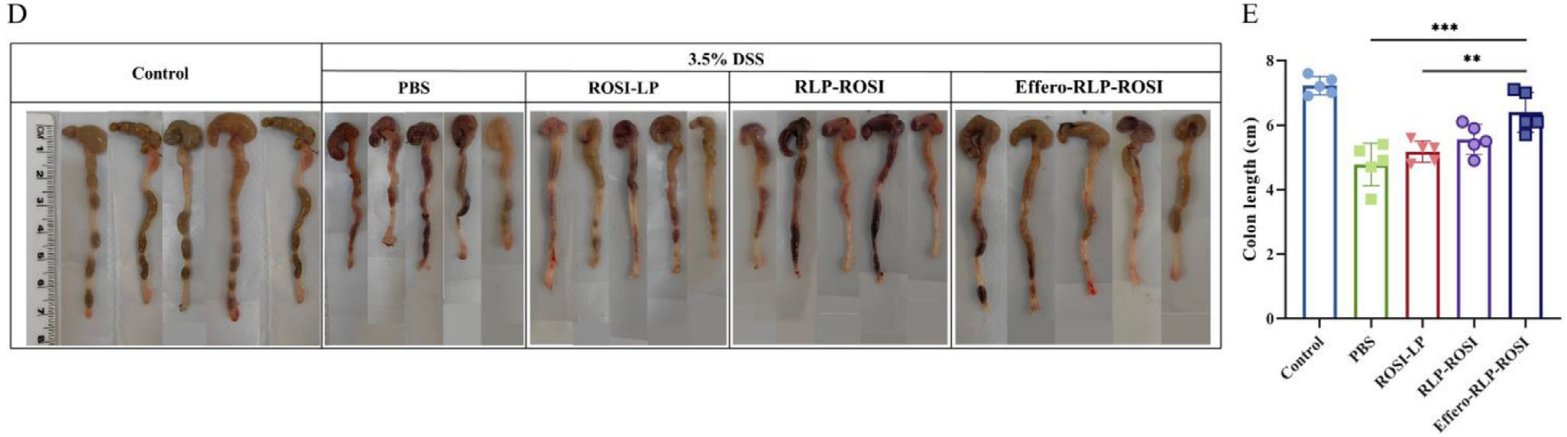


The duplicated images were replaced with correct images in the revised figure below:


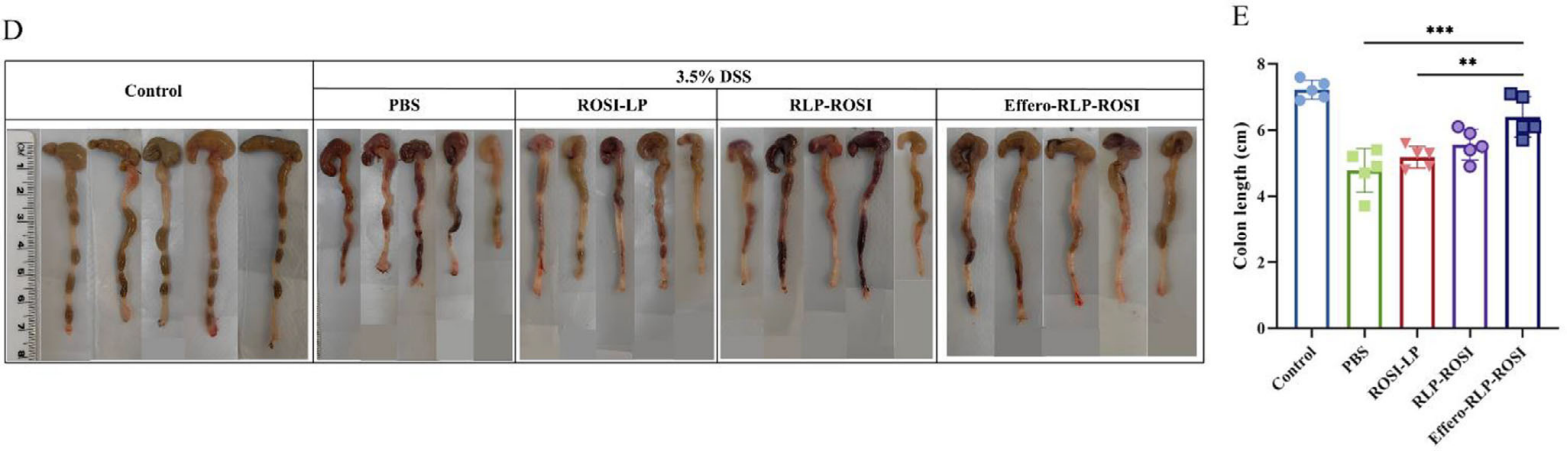


We apologize for this error.

## Supporting information



Supporting Information

