# Supplementary material for: Synthetic Biomimetic Liposomes Harness Efferocytosis Machinery for Highly Efficient Macrophages‐Targeted Drug Delivery to Alleviate Inflammation
Source: Adv Sci (Weinh). 2024 May 24;11(29):2308325. doi: 10.1002/advs.202308325 (PMC11304272; doi:10.1002/advs.202308325)
Supplement: Supplementary file 1 — Supporting Information [file ADVS-11-2308325-s001.pdf]

ADVANCED SCIENCE
Open Access

Supporting Information

for *Adv. Sci.*, DOI 10.1002/advs.202308325

Synthetic Biomimetic Liposomes Harness Efferocytosis Machinery for Highly Efficient Macrophages-Targeted Drug Delivery to Alleviate Inflammation

Run Han, Zhengyu Ren, Qi Wang, Haidong Zha, Erjin Wang, Mingyue Wu, Ying Zheng and Jia-Hong Lu**

Supporting Information

Synthetic biomimetic liposomes harness efferocytosis machinery for highly efficient macrophages-targeted drug delivery to alleviate inflammation

Run Han[#], Zhengyu Ren[#], Qi Wang, Haidong Zha, Erjin Wang, Mingyue Wu, Ying Zheng*, Jiahong Lu*

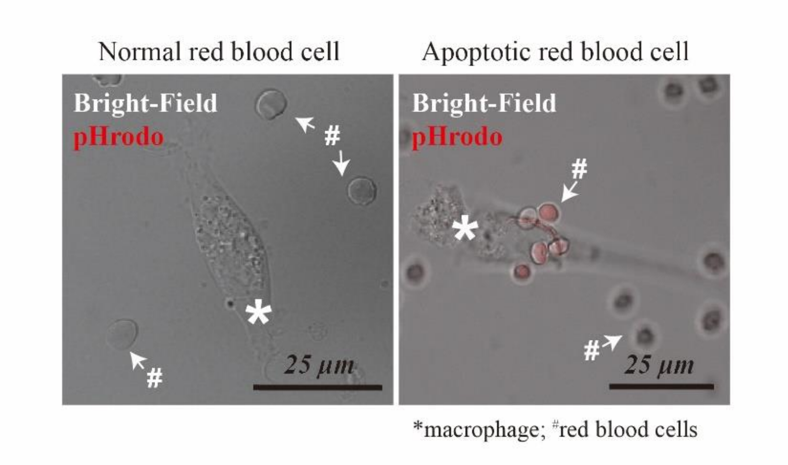

Fig. S1. The phenomenon of efferocytosis that apoptotic red blood cell endocytosed by macrophages.

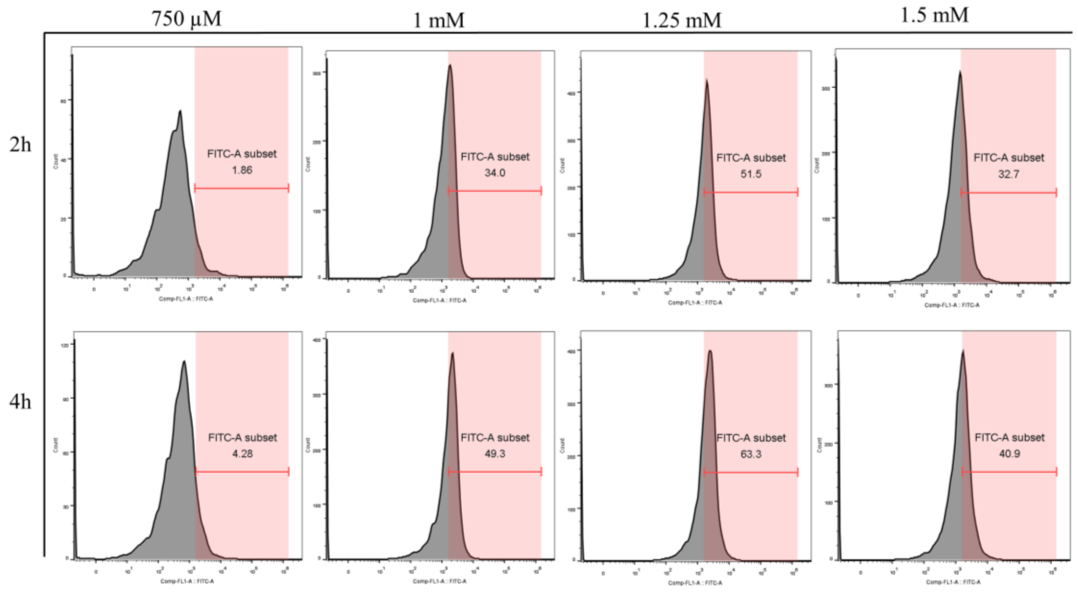

Fig. S2. The percentage of apoptotic RBC with series concentration of H_2O_2 treatment with different time.

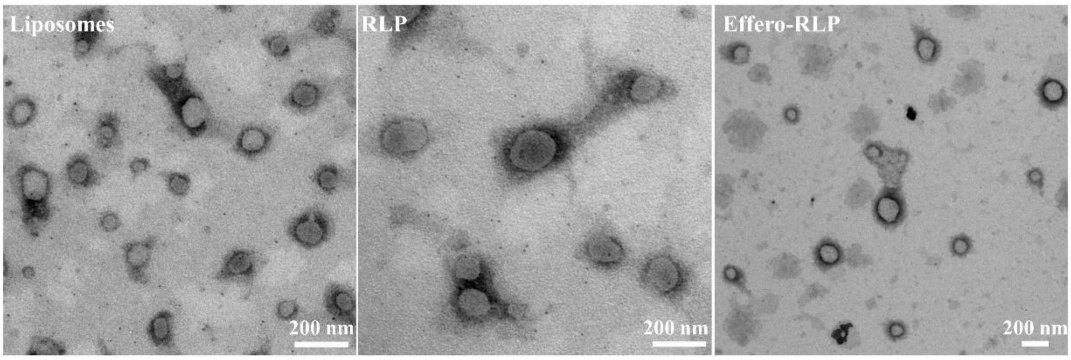

Fig. S3. The TEM images of liposomes, RLP and Effero-RLP.

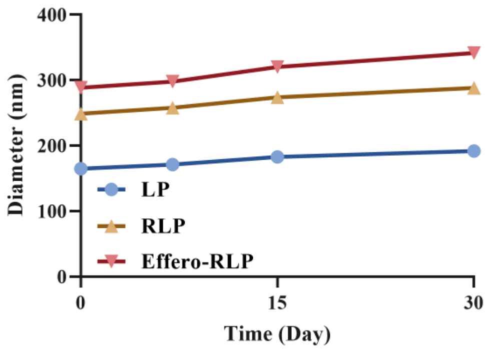

Fig. S4. The stability of liposome, RLP, Effero-RLP for 30 days.

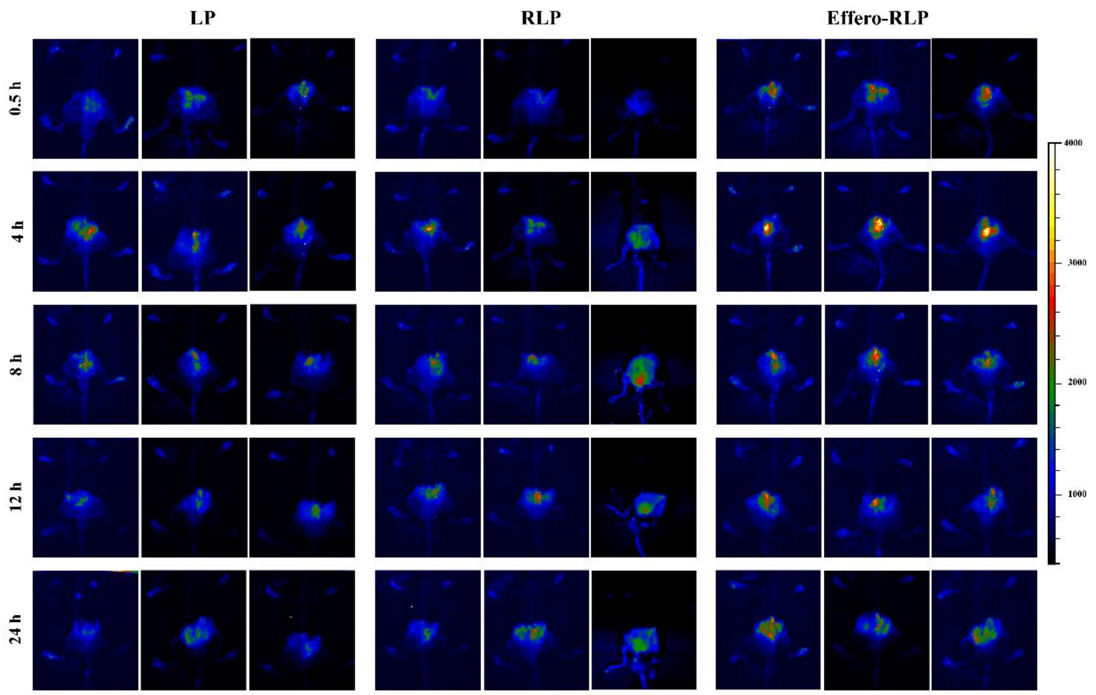

Fig. S5. Biodistribution of DiD labeled LP, RLP and Effero-RLP on colitis mouse after injection for 24 h.

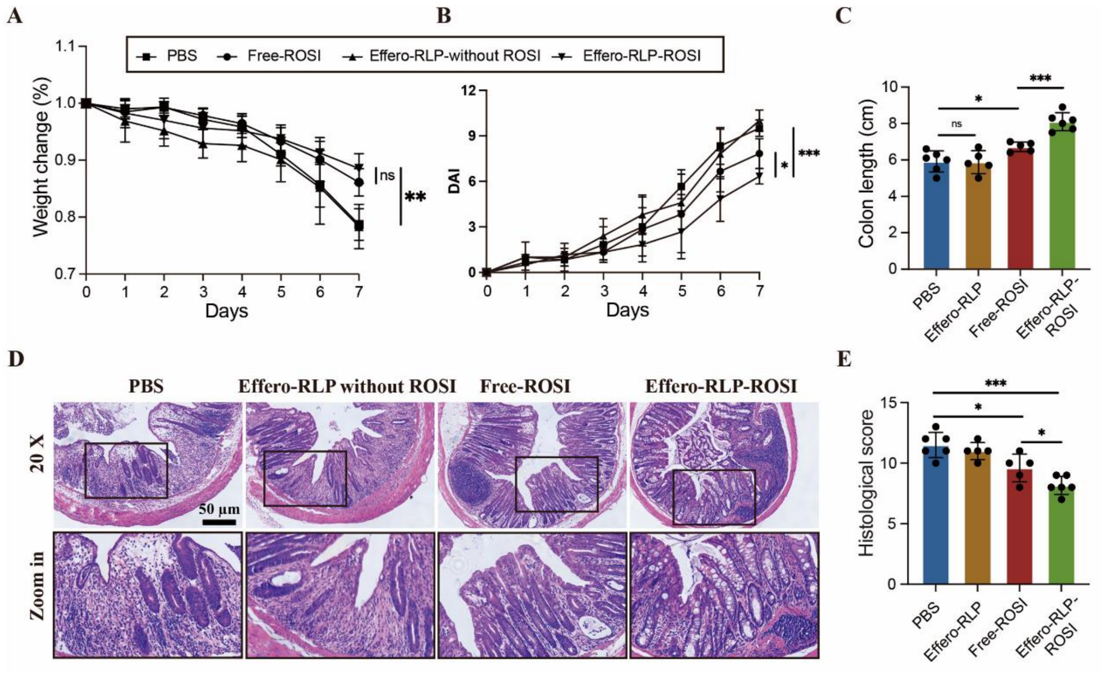

Fig. S6. The treatment effect of biomimetic liposome carrier without drug, free ROSI and Effero-RLP-ROSI. (A) The body weight change of each mouse in different groups. (B) The DAI score of each mouse in different treatment groups. (C) The colon length in different treatment groups. (D) The hematoxylin-eosin (H&E) staining images of colons tissues in different treatment groups. (E) The colonic histological damage score for HE staining images evaluation. Data were represented as mean ± SD, n=5. All significant differences were determined by one-way ANOVA followed

by Tukey's honestly significant difference post-hoc test (ns: no statistically difference, *p <0.05, **p <0.01, ***p <0.001.

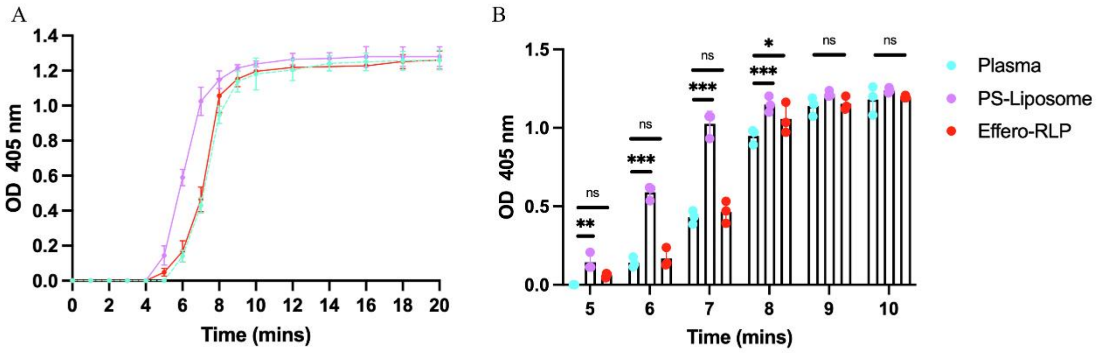

Figure S7. Comparation of coagulation-inducing ability of Effero-RLP and PS-loaded liposome by Overall Hemostatic Potential (OHP) assay. (A) Differences in OHP assay curves of normal condition and with the treatment of Effero-RLP or PS-loaded liposome. (B) Quantification of OD values at different time points. Data were represented as mean ± SD, n=3. All significant differences were determined by one-way ANOVA followed by Tukey's honestly significant difference post-hoc test (ns: no statistically difference, *p <0.05, **p <0.01, ***p <0.001.
