# Supplementary material for: Correction to [Synthetic Biomimetic Liposomes Harness Efferocytosis Machinery for Highly Efficient Macrophages‐Targeted Drug Delivery to Alleviate Inflammation]
Source: Adv Sci (Weinh). 2025 Aug 22;12(36):e15155. doi: 10.1002/advs.202515155 (PMC12462911; doi:10.1002/advs.202515155)

**Supporting information**

Erratum in manuscript “Synthetic Biomimetic Liposomes Harness Efferocytosis Machinery for Highly Efficient Macrophages-Targeted Drug Delivery to Alleviate Inflammation” (DOI: <https://doi.org/10.1002/advs.202308325>)

Upon reviewing our published work, we identified an inadvertent error in Figure 5D on page 2308325 (page 8 of 16). Specifically, In the control group and the RLP-ROSI group, one colon tissue image in each group was mistakenly shown two times (the second and fifth images in control group, and the third and fifth images in RLP-ROSI group). After thoroughly reviewing the original raw data, the duplicated images were replaced with correct images in the revised figure. The details and corresponding original data have been labeled in the figure below.


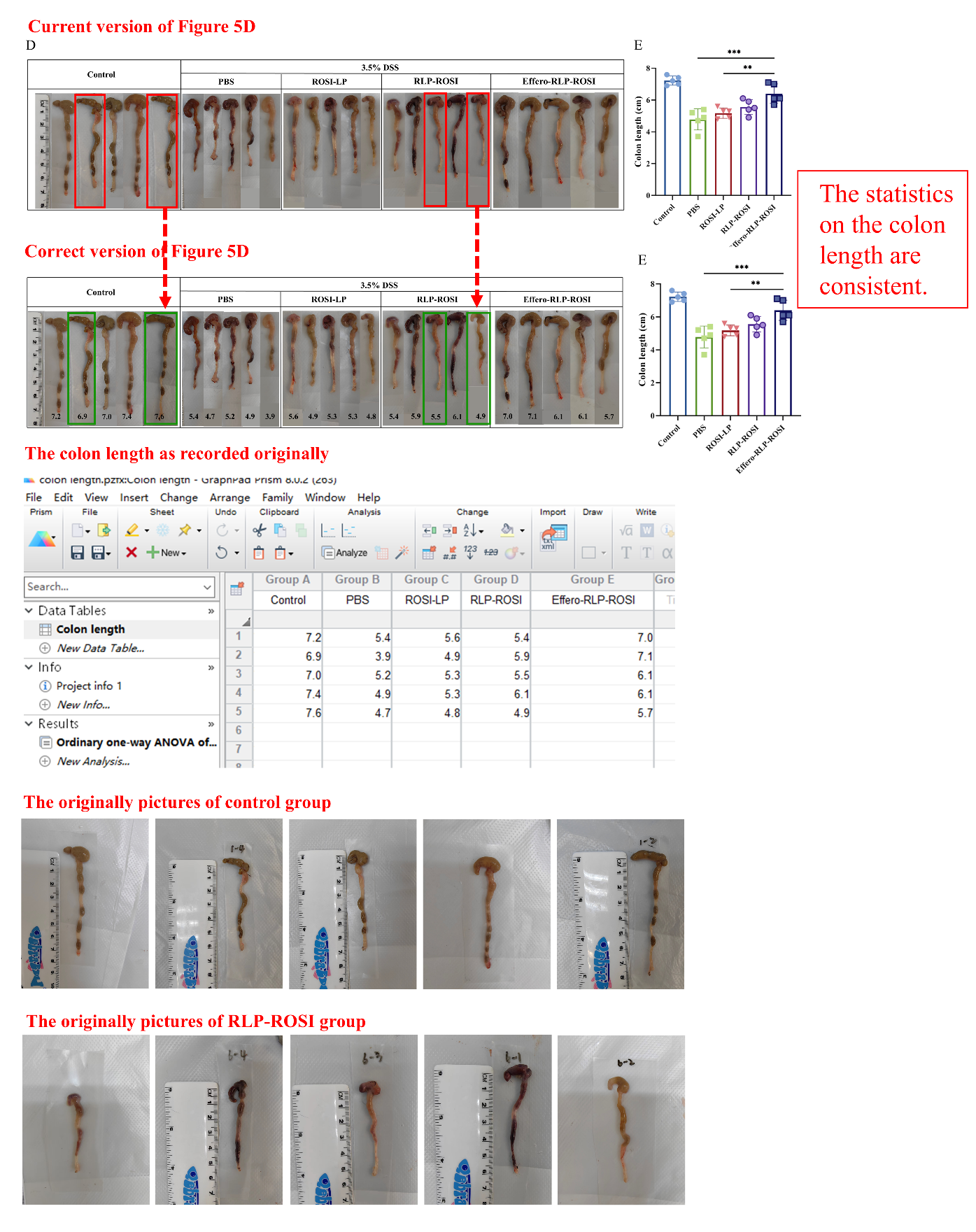


**Revised data**


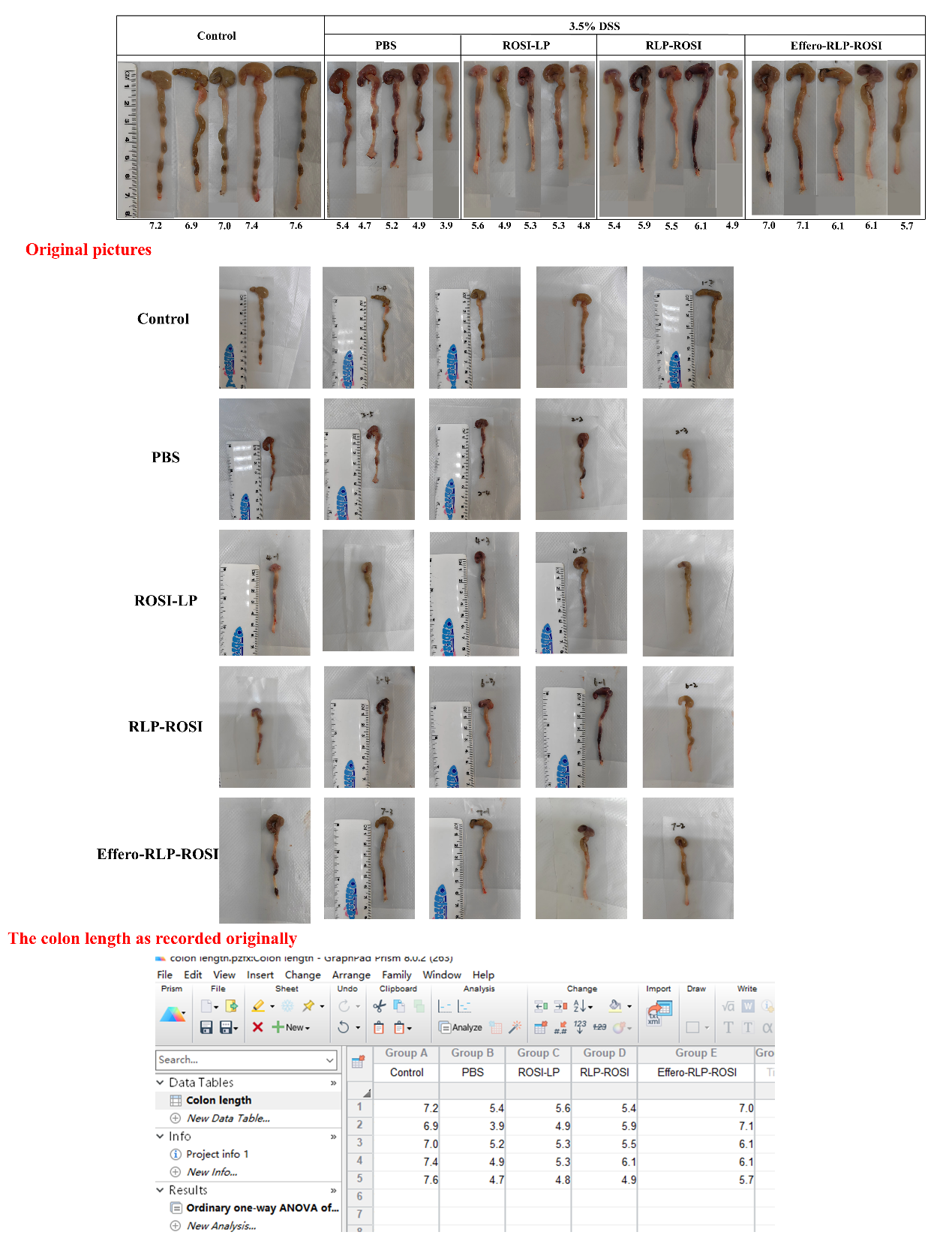


**Correct Figure 5**


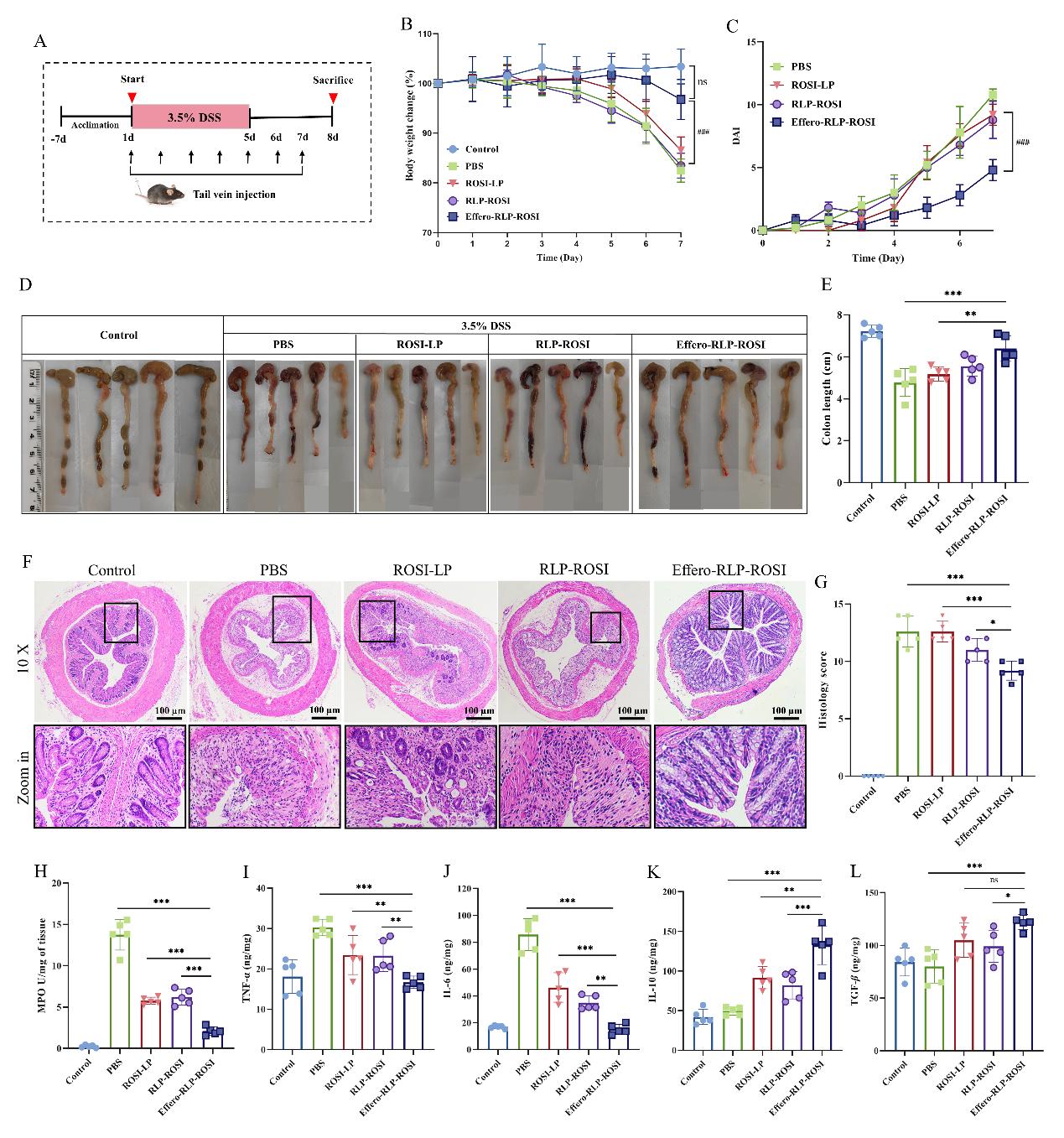

Supplement: Supplementary file 1 — Supporting Information [file ADVS-12-e15155-s001.docx]
